# Exploring Dynamic Changes in Microbial Succession and Flavor-Active Metabolites During Pickled and Dried Mustard Fermentation of Traditional Fermented Vegetables

**DOI:** 10.3390/foods15193486

**Published:** 2026-09-29

**Authors:** Xue Jiang, Hong Xu, Huang Zhou, Mingyong Xie, Tao Xiong, Zhanggen Liu

**Affiliations:** 1 State Key Laboratory of Food Science and Resources, Nanchang University, No. 235 Nanjing East Road, Nanchang 330047, China; 2 College of Food Science and Technology, Nanchang University, No. 235 Nanjing East Road, Nanchang 330031, China; 3 International Institute of Food Innovation International, Nanchang University, Luozhu Road, Xiaolan Economic and Technological Development Zone, Nanchang 330200, China; 4 Ji’an College, 133 Ji’an South Avenue, Ji’an City Center, Ji’an 343000, China

**Keywords:** lactic acid bacteria, physicochemical characteristics, flavor formation, microbial succession, fermentation process, core microorganisms

## Abstract

Pickled and dried mustard (PDM) is a traditional dish of the Hakka people that is well-known in many parts of the world. However, past scientific studies on PDM provide little information on the relationship between the microbiome composition of the PDM fermentation process and flavor formation, which restricts the industrialization and improved quality control of PDM. Therefore, this study investigated the microbiome composition, flavor metabolites, physicochemical properties, and the relationships among them in PDM from Shaoxing. During fermentation, the main aromas of PDM are similar to pine and orange oils, with a strong fat aroma, and are mainly contributed by dodecanal in the early and middle stages of fermentation. As the fermentation progressed, the phenolic and aldehyde contents increased markedly; the major aldehydes in PDM were dodecanal, nonanal, and phenylacetaldehyde. The microbial succession during PDM fermentation was a gradual transition from *Weissella* sp. and *Basidiomycota* to *Latilactobacillus*, *Lactiplantibacillus*, and *Ascomycota*. We identified candidate core microorganisms potentially related to characteristic flavor development in PDM: *Debaryomyces* sp., *Weissella cibaria*, *Lactiplantibacillus pentosus*, *Weissella* sp. *NBRC 107272*, *Latilactobacillus sakei*, *Cryptococcus* sp., *Filobasidium magnum*, *Cystofilobasidium macerans*, *Weissella hellenica*, *Levilactobacillus brevis*, and *Weissella* sp. *NBRC 107245*. The findings of this study will be an important guide to optimizing the production process and quality of PDM.

## 1. Introduction

Pickled and dried mustard (PDM, or Meigan cai), a fermented leaf mustard product, is a well-known Chinese vegetable preserve. Its pleasant taste, rich nutrients, and two-year shelf life at room temperature have made it increasingly favored by consumers. Typically stir-fried with meat or other dishes, it is widely eaten by all demographics. Previous studies on PDM have mainly focused on chemical and sensory aspects, including changes in phenolic acids and antioxidant activities during pickling [1], the effects of different cooking methods on volatile compounds and antioxidant properties [2], and the analysis of volatile components during PDM fermentation [3]. Although these studies provided useful information on flavor-related chemical changes, they did not investigate the microbial succession during PDM fermentation or its relationship with flavor formation. The microbial community structure of PDM and the relationship between microbial composition and flavors are not clear, hampering the market development, industrialization, and quality improvement of PDM. Therefore, elucidating the microbial succession and its relationship with flavor formation in PDM is scientifically important for understanding the microbial mechanisms underlying the characteristic flavor of traditional spontaneously fermented vegetables, and practically important for guiding process control, quality standardization, and industrial production of PDM.

A detailed understanding of the flavor profiles of fermented vegetables is vital for the evaluation and improvement of quality control to maintain the flavor quality of PDM. Volatile flavor compounds (VFCs), free amino acids (FAAs), organic acids (OAs), and sugars are among the many important contributors to the flavor profile of fermented vegetables and to the richness of the taste and aroma of PDM. VFCs, including aldehydes, alcohols, esters, and sulfur-containing compounds, are the major determinants of the characteristic aroma of fermented vegetables and are largely generated through microbial metabolism of lipids, carbohydrates, and amino acids [4,5]. FAAs contribute directly to taste perception, with sweet, umami, and bitter amino acids shaping the overall taste profile; they also serve as precursors for the formation of aroma-active compounds such as aldehydes and higher alcohols [4]. OAs, particularly lactic and acetic acids, are the primary taste-active compounds responsible for the characteristic sourness of fermented vegetables and also contribute to flavor development by modulating the metabolic activity of fermenting microorganisms [6,7]. Sugars serve as the main energy substrates for lactic acid bacteria (LAB) and other fermenting microorganisms, and their metabolism leads to the production of organic acids, alcohols, and other flavor compounds that collectively shape the final flavor profile [8]. In PDM, glucosinolate degradation products and aldehydes have been identified as the dominant volatile compounds [9], while the dynamic changes in these flavor-related metabolites during fermentation remain to be systematically investigated.

Researchers widely apply metabolomics to investigate the flavor of fermented foods; 15 key chemical components have been identified in PDM using metabolomics. The volatile compounds in PDM were mainly aldehydes, acids, and esters, with aldehydes being the most diverse. In addition, although isothiocyanates and nitriles were present at low concentrations, they are derived from glucosinolate degradation and contribute importantly to the characteristic flavor of PDM [10]. To explore microbial community shifts during vegetable fermentation, high-throughput gene sequencing is frequently used [11,12]. Changes in metabolite concentrations during vegetable fermentation are associated with specific microbial metabolic pathways [13,14]. The microbial community structure significantly shapes the flavor development of fermented vegetables, and lactic acid bacteria (LAB) are consistently reported to dominate in traditional spontaneously fermented vegetables [15,16,17]. Moreover, *Lactobacillus* significantly contributes to flavor improvement, shelf-life extension, and safety enhancement in fermented vegetables [18,19].

Despite extensive research on factors such as pH and nitrite affecting fermented vegetable microbiomes [20,21], studies exploring the links between metabolites and PDM microbiome composition are scarce. In other fermented vegetables, microbial succession has been well documented. In paocai and suancai, the bacterial community shifts from *Leuconostoc*-clade dominance to *Lactobacillus*-clade dominance, which is closely associated with organic acid accumulation and flavor development [22,23]. In Zhacai, halophilic bacteria and LAB dominate after 30 days, with *Debaryomyces* increasing as fermentation progresses [24]. A recent review on mustard fermentation summarized a common pattern: *Enterococcus* and *Leuconostoc* dominate early, lactic acid cocci and facultative anaerobic LAB dominate mid-fermentation, and homolactic LAB dominate late, with acid production, oxygen consumption, and pH changes as key drivers [6]. However, the specific microbial succession and its relationship with flavor formation in PDM remain unclear, which is the focus of the present study.

Therefore, this study aimed to integrate physicochemical, flavor compound, and high-throughput sequencing analyses to reveal the relationship between microbial succession and key flavor formation during PDM fermentation and to identify the candidate core microorganisms potentially related to flavor formation and key flavor compounds.

## 2. Materials and Methods

### 2.1. Chemical Standards

Sucrose, glucose, fructose, lactic acid, acetic acid, citric acid, and malic acid were obtained from Aladdin (Shanghai, China). All amino acid reagents were obtained from Solarbio (Beijing, China). Other chemicals were obtained from Aladdin (Shanghai, China).

### 2.2. Preparation and Sampling

Twenty-eight homemade PDM samples were collected from four fermentation jars at seven time points (before sealing and days 1, 3, 5, 7, 10, 13) under identical conditions in Shaoxing, Zhejiang, China. PDM was prepared by pickling potherb mustard with salt (25:1), tightly packing it into jars, and naturally fermenting at room temperature (20–25 °C) for two weeks. Samples from the upper, middle, and lower layers of each jar were pooled and stored at −80 °C.

### 2.3. Physical and Chemical Analysis

A salinometer (SA287, Qingdao Tridonic, Qingdao, China) and a pH meter (Mettler Toledo, Shanghai, China) were used for salinity and pH measurements, respectively. The methods for determining titratable acidity (TA) and nitrite content were based on the study by Zhao et al. [25].

### 2.4. Analysis of OAs, Reducing Sugars, and FAAs

OAs and sugars were quantified using HPLC (1260 Infinity II, Agilent Technologies, Santa Clara, CA, USA) following Zhao et al. [25]. Quantification was performed using the external standard method [26]. FAAs were analyzed using an automatic amino acid analyzer (S-433D, Sykam, Munich, Germany) [25]. The threshold values for amino acids were obtained from Hu et al. [27].

### 2.5. Analysis of VFCs

PDM volatiles were profiled via gas chromatography-quadrupole-time-of-flight mass spectrometry (HS-SPME-GC–QTOF/MS; 7890B GC, 7250 Q-TOF/MS, Agilent) with slight modifications to the published method [28]. A 5 mL sample was extracted with a 50/30 μm DVB/CAR/PDMS fiber at 60 °C for 20 min and then desorbed at 250 °C for 5 min. GC conditions: DB-WAX column (60 m × 250 μm × 0.25 μm); oven program: 40 °C held for 2 min, then raised to 170 °C at 5 °C/min and held for 2 min, then raised to 250 °C at 10 °C/min and held for 5 min; inlet 250 °C; split ratio 10:1. MS: full scan, EI (70 eV, 250 °C), m/z 29–350. Identification used the NIST 17.0 library (MS and RI matching). Retain compounds with a match score greater than 75. ROAVs were calculated following [29,30]. Odor thresholds were sourced from Gemert et al. [31].

### 2.6. High-Throughput Sequencing Analysis

Microbial composition of PDM was analyzed using third-generation sequencing. Genomic DNA was extracted with an OMEGA kit (D5625-01) according to the manufacturer’s instructions. For bacterial and fungal community analysis, full-length 16S rRNA and ITS rRNA genes were amplified using primers 27F (5′-AGRGTTTGATYNTGGCTCAG-3′)/1492R (5′-TASGGHTACCTTGTTASGACTT-3′) and ITS-1F (5′-CTTGGTCATTTAGAGGAAGTAA-3′)/LR3 (5′-CCGTGTTTCAAGACGGG-3′), respectively, and then barcoded for SMRT sequencing [32]. Sequencing was performed on a PacBio Sequel platform (Shanghai Personal Biotech). Raw reads were quality-filtered with SMRT Link (v5.0.1.9585) and processed using QIIME (v1.8.0) [33]. Reads with exact barcode matches were assigned to individual samples, chimeras were removed with DADA2 to generate amplicon sequence variants (ASVs) [34], and taxonomic assignment was performed via blastn (BLAST+ version 2.13.0) against the NCBI nt database.

### 2.7. Identification of LAB

The LAB were isolated and identified according to our previous studies [35].

### 2.8. Data Analysis

Results are means ± SD. Microbiome comparisons were assessed by the Kruskal-Wallis test (*p* < 0.05) and visualized with GraphPad Prism 8.0 and Origin 2023. VFC data were processed via SIMCA 14.0 (Umetrics AB, Umea, Sweden) for principal components analysis (PCA) and orthogonal partial least-squares discriminant analysis (OPLS-DA). Heatmaps, canonical correlation analysis (CCA), and Spearman correlations were generated on Genescloud (https://www.genescloud.cn) (accessed on 22 August 2026).

## 3. Results and Discussion

### 3.1. Physical and Chemical Analysis

**Figure 1:**
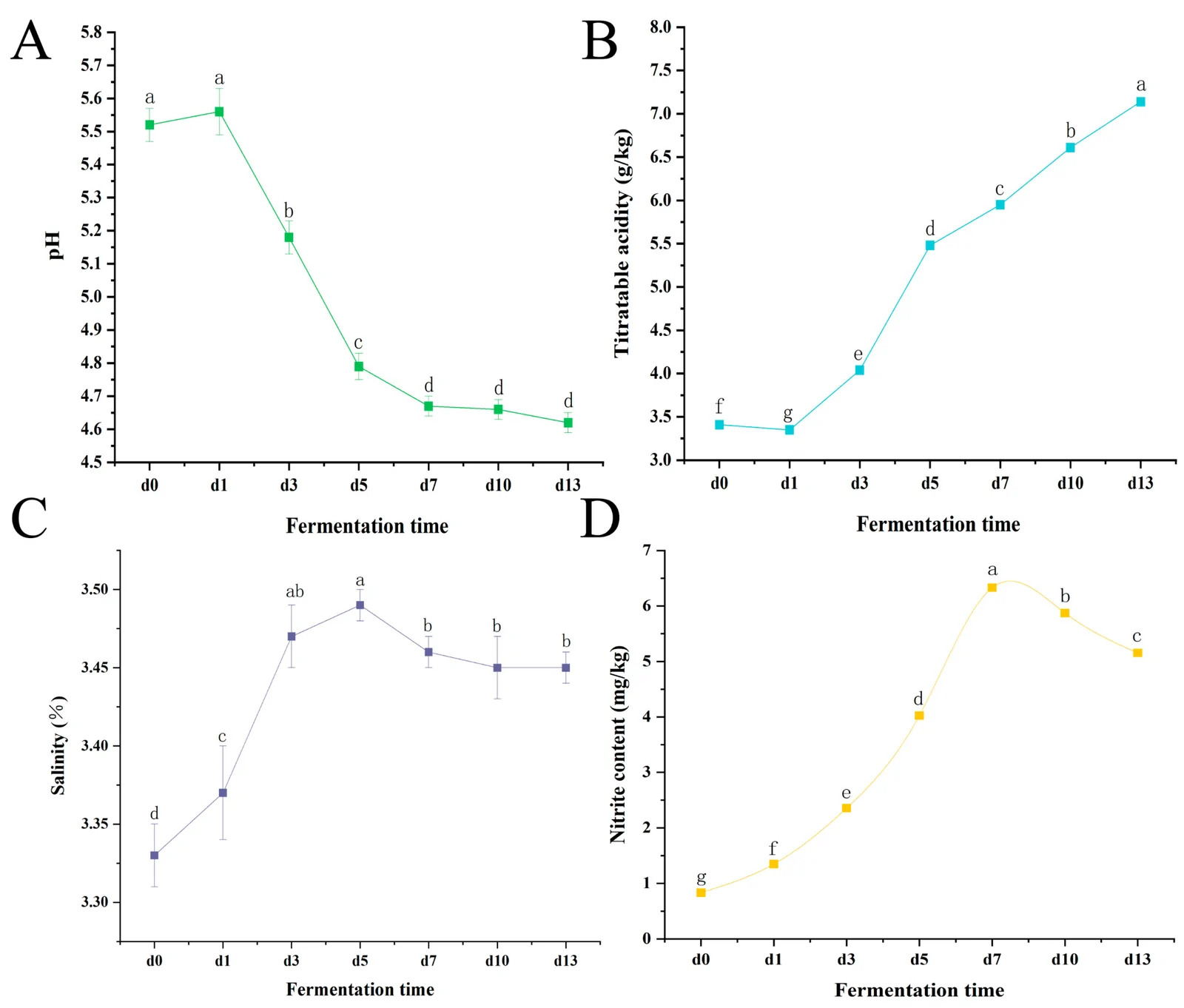
The physicochemical properties of PDM during natural fermentation. (**A**) pH. (**B**) Titratable acidity. (**C**) Salinity. (**D**) Nitrite content. Different letters indicate significant differences among fermentation time points (*p* < 0.05).

During PDM fermentation, pH decreased over time. It started at 5.5, fell sharply in the early phase, and leveled off at 4.6 by the end (Figure 1A). The TA increased from 3.4 g/kg at day 0 of fermentation to 7.1 g/kg at day 13 (Figure 1A,B). This may be ascribed to the higher content of lactic and acetic acids [36]. Indicating that these fermented vegetables are mature PDM [37]. However, salinity did not change significantly during fermentation and remained essentially stable at 3.45% by the middle stage of fermentation (Figure 1C). Nitrite content first increased and then decreased, which may be related to nitrite reduction by LAB [38,39] or acid-mediated nitrite degradation under the acidic fermentation environment [40]. The nitrite content ranged from 0.83–6.33 mg/kg (Figure 1D), which is within the requirement of the Chinese standard, GB 2762-2025 (<20 mg/kg) [41].

### 3.2. Changes in Sugar and Organic Acid (OA) Concentrations During Fermentation

**Figure 2:**
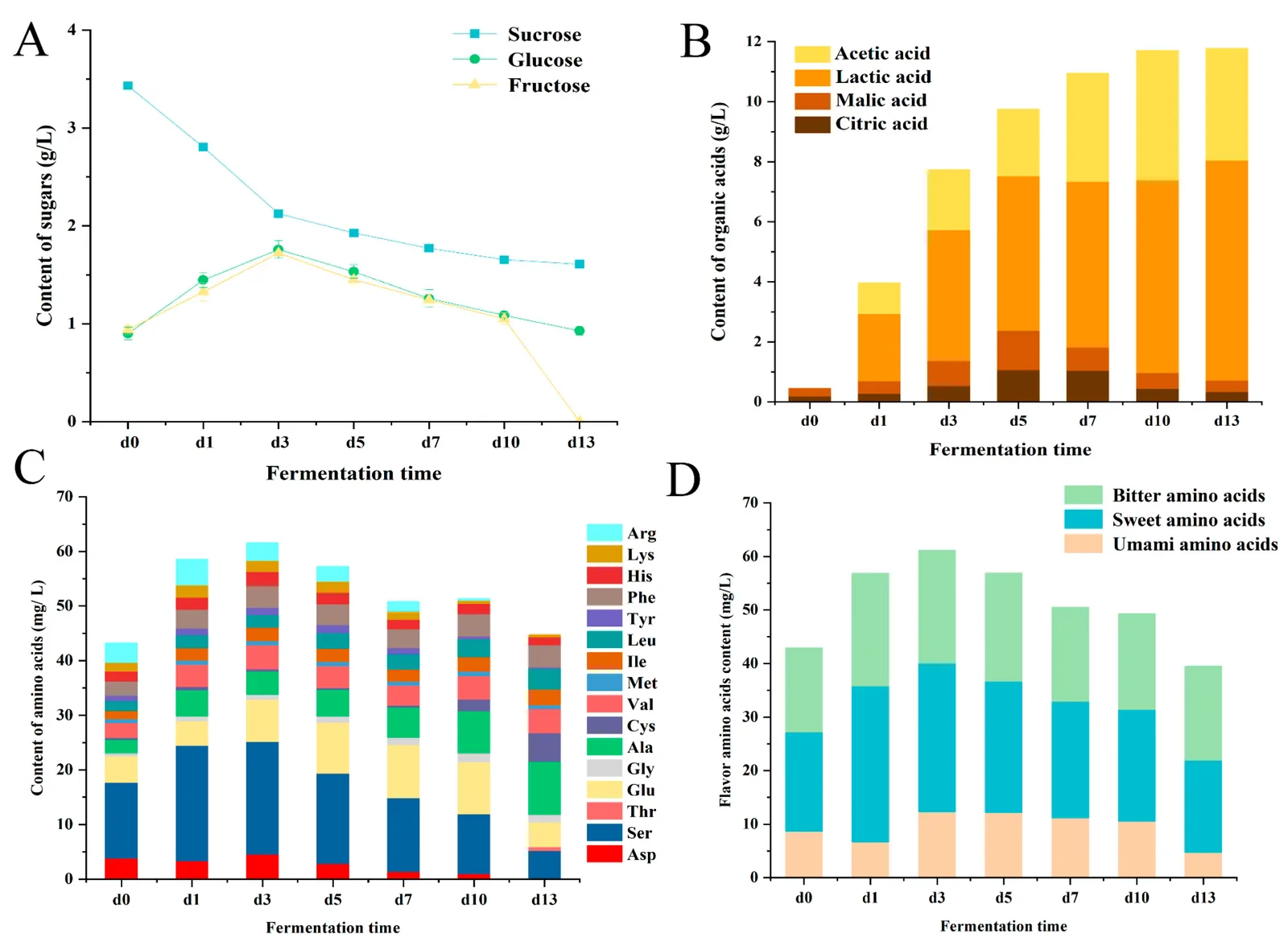
The non-volatile flavor substances of PDM during natural fermentation. (**A**) Sugars. (**B**) Organic acids. (**C**,**D**) Free amino acids.

Reducing sugars are major energy substrates for the PDM microbial community. Their total content decreased throughout fermentation, probably because LAB utilized carbohydrates from the vegetables and transformed them into organic acids [42]. The sucrose concentration gradually decreased with time, with a relatively rapid decrease during the pre-fermentation period. Glucose and fructose rose during the first three days, then declined, with concentrations remaining below 2 g/L throughout (Figure 2A). The early rapid decrease in sucrose and transient increase in glucose and fructose may be attributed to invertase activity of dominant microorganisms such as *Lactobacillus*, which hydrolyze sucrose into glucose and fructose [43]. The rapid depletion of fructose from day 10 to day 13 may be explained by carbon catabolite repression and preferential fructose utilization by late-stage LAB [43] and yeasts (e.g., *Debaryomyces*) [44].

As sugars are transformed into acids during fermentation [45], total OAs progressively increased. Lactic and acetic acids, the main LAB metabolites in fermented vegetables, are primarily generated via the glycolytic and phosphoketolase pathways [23], and were produced relatively rapidly from the first day, then peaked on the 13th and 10th days, respectively (Figure 2B). Meanwhile, the constant TA increase during PDM fermentation resulted from the buildup of lactic and acetic acids. Taste threshold evaluation indicated that these four acids were the key taste-active components (TAV > 1), which imparted the characteristic sourness of PDM (Table S1 [46]) and contributed to pathogen inhibition [47].

### 3.3. Changes in FAAs Composition During Fermentation

Seventeen FAAs were found in the PDM fermentation, which were divided into three different types: namely, sweet, umami, and bitter amino acids (Table S2 [48]). The total FAA content increased rapidly from 0–3 days, then slowly decreased from 3 to 13 days (Figure 2C). The initial increase in FAA content presumably resulted from microbial biosynthesis and transamination of OAs, and the later decrease appeared to result from consumption by microbial metabolism as a nutrient source [49]. Previous studies reported that Ser, Glu, and Ala were the main FAAs in fermented vegetables, such as Chinese bamboo shoots, suancai, and radish paocai [42,50], and the results of this study were similar. Sweet amino acids rose initially, then fell by mid-fermentation (Figure 2D), while umami amino acids remained stable until the end. Bitter amino acids increased sharply in the early stage and decreased in the middle. Compared to raw materials, threonine, alanine, and cysteine increased in fermented PDM, with cysteine showing the greatest rise (16.4-fold). Although bitter amino acids dominated the FAA profile, their bitterness was partially masked by salinity and acidity, as shown by sensory evaluation [51]. Moreover, FAAs in mature PDM did not act as key taste-active compounds (TAV > 1, Table S2 [48]).

### 3.4. Measurement of Differential VFCs During Fermentation

**Figure 3:**
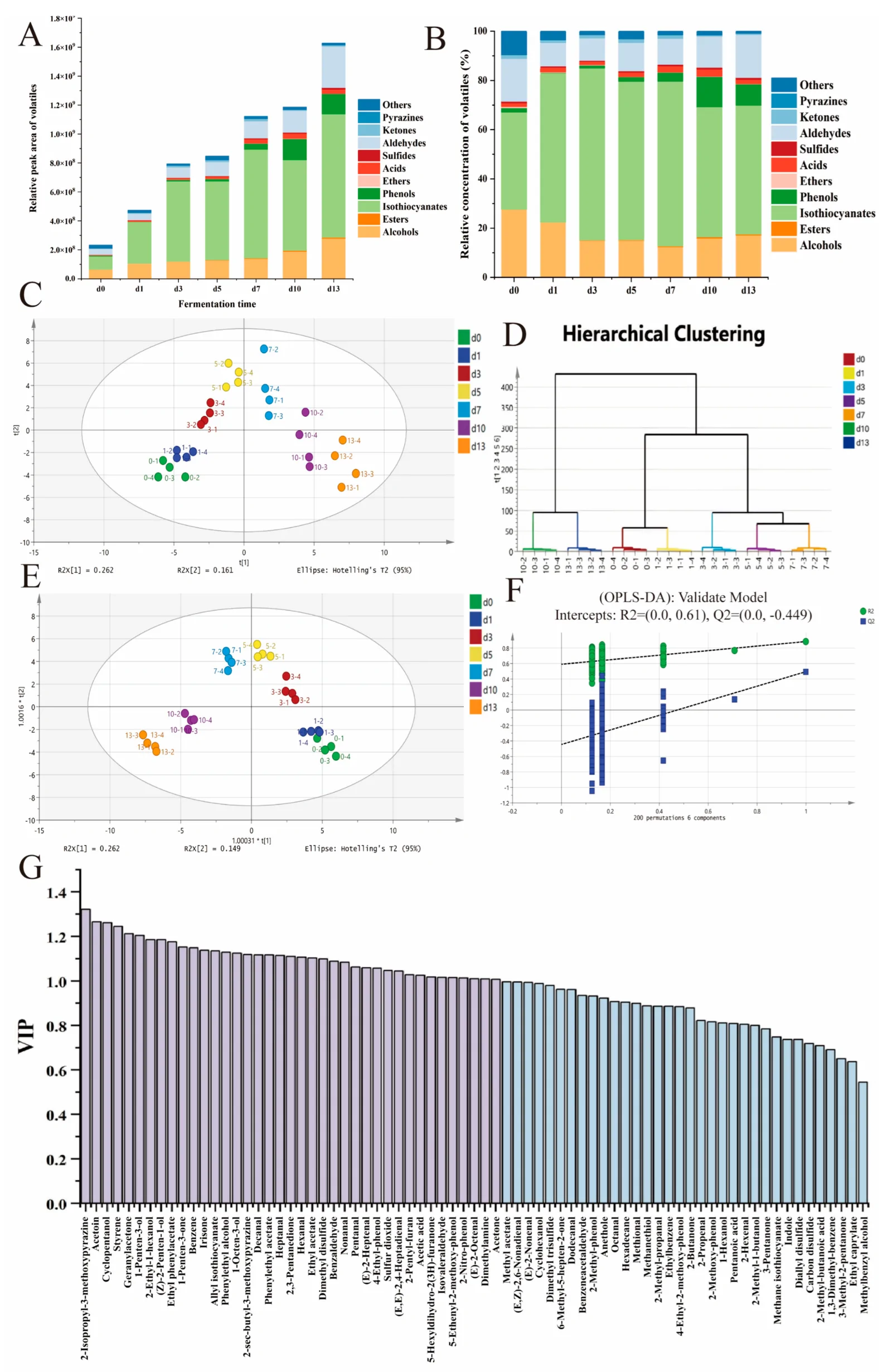
Dynamic changes in volatile flavor during PDM fermentation. The peak areas (**A**) and relative concentrations (**B**) of different types of volatile components. (**C**) The scatter plot of principal component analysis (PCA). (**D**) Hierarchical clustering analysis. (**E**) Orthogonal partial least squares discriminant analysis (OPLS-DA) plot. (**F**) Validation plots of the OPLS-DA model. (**G**) Variable importance for the projection (VIP) plot.

In total, 74 kinds of VFCs were identified, namely nineteen aldehydes, eleven alcohols, ten ketones, six esters, six phenols, five aromatics, five sulfides, three acids, three heterocyclic compounds, two isothiocyanates, one sulfur oxide, one amine, one alkane, and one ether (Table S3). The total peak area of VFCs increased with fermentation time, and the main aromas were “pine leaf oil”, “orange oil”, and “fat”, mainly contributed by dodecanal in the early and middle stages of fermentation (Figure 3A,B). In addition, there were “pea” aromas from pyrazines. In the later stages of fermentation, the nonanal and phenylacetaldehyde contents increased, contributing “dried plum” and “citrus” aromas for the PDM.

Compounds with 1 ≤ ROAV ≤ 100 are considered to be key volatile compounds [52]. Comparison of ROAVs identified 26 aroma-active compounds, including eleven aldehydes, four phenols, four alcohols, two pyrazines, two sulfides, one ester, one ketone, and one isothiocyanate (Table S4). Of these aroma-active compounds, aldehydes served as the key odorants in PDM, which were mainly generated by amino acid Strecker degradation and thermal degradation [53,54]. The major aldehydes in PDM are dodecanal (soapy, waxy, aldehydic, citrus, orange, rind, floral), nonanal (waxy, aldehydic, citrus, fresh, green, lemon peel, cucumber, fatty), phenylacetaldehyde (green, floral, rose, clover), and heptanal (green, aldehydic, oily, cortex, grassy, clover, cilantro) (http://www.perflavory.com/) (accessed on 22 January 2026). Nonanal and heptanal derive from the degradation of unsaturated fatty acids [53]. The biosynthesis of phenylacetaldehyde was linked to phenylalanine [52]. These aldehydes are also major aroma components in many other fermented foods, such as fermented cucumbers [55] and paocai [49,56].

Isothiocyanates usually have a strong pungent “mustard” flavor and are mainly glucosinolate hydrolysates in cruciferous vegetables [57]. The most representative isothiocyanate in PDM was allyl isothiocyanate, which is reported to be further hydrolyzed into 3-butenenitrile, carbon disulfide, and dimethyl disulfide as fermentation progresses [58], and is also widely found in suancai [45,59]. Pyrazine compounds are mainly derived from the Maillard reaction. Although 2-isopropyl-3-methoxypyrazine (pea, green pea, earthy, beany, chocolate, nutty) and 2-*sec*-butyl-3-methoxypyrazine (musty, pea, green pea, galbanum, pepper, bell pepper) (http://www.perflavory.com/) had low relative contents, but still had a noticeable effect on the overall PDM aroma (ROAVs of 98.92 and 80.92, respectively) because of their ultra-low aroma thresholds [60,61].

High ROAVs were observed for dimethyl disulfide, carbon disulfide, and methanethiol in PDM. These compounds likely originate from the primary breakdown products of glucosides present in fresh raw vegetables [56,58]. These sulfur compounds were also important flavor components of fermented vegetables, such as paocai [62], Chinese cabbage, cabbage, and white radish [49].

Fermentation led to a significant increase in phenolics and aldehydes. Furthermore, the majority of aroma-active compounds were produced chiefly during the middle and final phases of PDM fermentation. PCA revealed a clear separation of samples according to fermentation time (Figure 3C). Hierarchical clustering analysis (HCA) was performed on the VFC profiles of PDM samples using Euclidean distance and Ward’s method. The samples from different time points were grouped into three clusters (days 0–1, 3–7, and 10–13; Figure 3D), indicating clear differences in aroma profiles as fermentation progressed.

OPLS-DA further distinguished VFC profiles across fermentation stages, with clear separation of PDM samples by time points (Figure 3E). Model reliability was confirmed by 200 permutation tests (The R^2^ and Q^2^ intercepts were 0.61 and −0.449; Figure 3F). Applying VIP ≥ 1.0 and *p* < 0.05 identified 23 differential volatile compounds (Figure 3G), 11 of which (nonanal, heptanal, decanal, benzaldehyde, 2-isopropyl-3-methoxypyrazine, 2-*sec*-butyl-3-methoxypyrazine, dimethyl disulfide, 4-ethylphenol, 5-ethenyl-2-methoxyphenol, 1-octen-3-ol and phenylethyl alcohol) are recognized as aroma-active in PDM. These compounds arose mainly in the mid-to-late fermentation, underscoring the marked dynamic changes in VFCs during PDM fermentation.

### 3.5. Microbiome Compositional Changes During Pickled and Dried Mustard Fermentation

**Figure 4:**
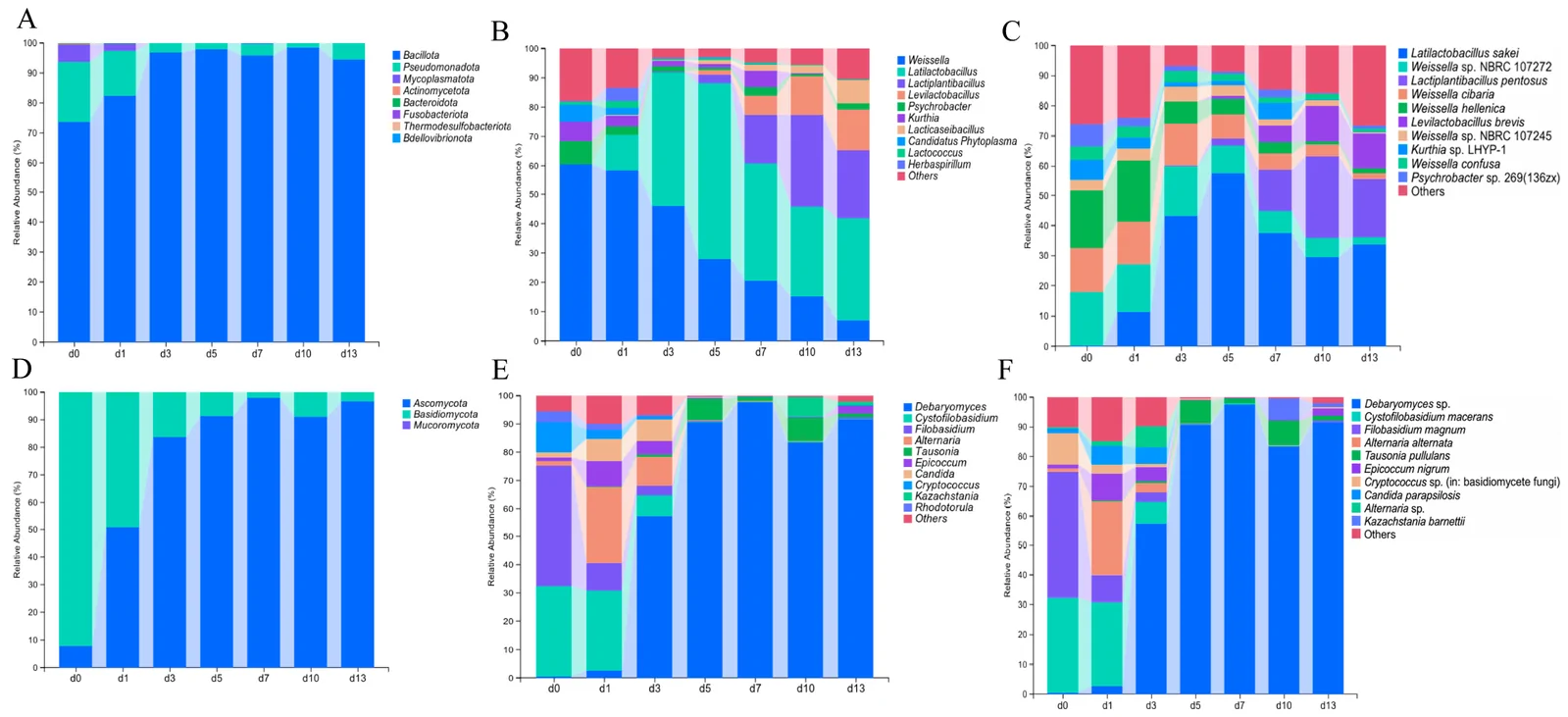
The relative abundance of microbial community composition in different fermentation stages at the phylum (**A**,**D**), genus (**B**,**E**) and species (**C**,**F**) levels.

At the phylum level, *Bacillota* (formerly Firmicutes) and *Pseudomonadota* were the dominant bacterial phyla, together accounting for an average relative abundance of 91.27%. *Bacillota* maintained a relative abundance above 70% throughout fermentation (Figure 4A), which is consistent with the fact that *Bacillota* was the dominant phylum in many other fermented vegetables, such as kimchi [63] and suancai [14]. At the genus level, the main microbial succession during PDM fermentation was a gradual transition from *Weissella* to *Latilactobacillus* and *Lactiplantibacillus* (Figure 4B), in agreement with previous findings, where they play a crucial role in the glycolysis pathway [50,64]. Moreover, at the species level (Figure 4C), *Latilactobacillus sakei*, *Weissella* sp. *NBRC 107272*, *Lactiplantibacillus pentosus*, and *Lactiplantibacillus brevis* were the dominant species. During the pre-fermentation period, *Weissella* sp. *NBRC 107272*, *Weissella cibaria*, and *Weissella hellenica* were the dominant species. Then, as the fermentation progressed and the pH decreased, the relative abundance of *Latilactobacillus sakei* increased, and it became dominant. Among these species, such as *Latilactobacillus sakei*, *Lactiplantibacillus pentosus*, *Lactiplantibacillus brevis*, and *Weissella cibaria*, have been used in many fermented foods and have been shown to improve flavor compounds [65,66,67].

The predominant fungal phyla in the PDM fermentation were *Ascomycota* and *Basidiomycota* (Figure 4D), which is consistent with other vegetable fermentations, such as Suancai [45]. *Ascomycota* was also the dominant fungal phylum at the middle and end stages of the PDM fermentation, with an average relative abundance of 74.18%. Overall, the fungal abundance shifted gradually from *Basidiomycota* to *Ascomycota* during PDM fermentation. *Debaryomyces* was the dominant fungal genus in the middle and later stages of fermentation (Figure 4E) and was also reported to be the dominant fungus in industrial Zhacai paocai, Sichuan radish paocai, and Northeastern sauerkrautother fermented vegetables [68,69,70]. The three most abundant fungal species were *Debaryomyces* sp., *Cystofilobasidium macerans*, and *Filobasidium magnum* (Figure 4F). Among them, *Cystofilobasidium macerans* and *Filobasidium magnum* mainly occurred in the early stage of fermentation. *Debaryomyces* sp. was the most abundant species at the middle and end stages of fermentation, which was different from a previous study in which *Debaryomyces hansenii* was the most abundant species in Zhaicai paocai and mustard green paocai [8,68]. This difference may be related to the type of fermented vegetable, which could shape the dominant yeast species.

### 3.6. Identification of Isolates and Species

A total of 92 LAB isolates were identifed (Table S5). 13 LAB species (Tables S5 and S6 and Figure S1) were identifed, including *Weissella cibaria* (25 isolates), *Weissella hellenica* (3 isolates), *Weissella* sp. (2 isolates), *Weissella confusa* (1 isolate), *Latilactobacillus curvatus* (2 isolates), *Lactobacillus* sp. (1 isolate), *Lactiplantibacillus plantarum* (28 isolates), *Loigolactobacillus coryniformis* (2 isolates), *Latilactobacillus sakei* (2 isolates), *Lactiplantibacillus paraplantarum* (1 isolate), *Loigolactobacillus coryniformis* subsp. *Torquens* (1 isolate), *Lactiplantibacillus argentoratensis* (1 isolate), and *Pediococcus pentosaceus* (23 isolates). Among these LAB species, *Weissella cibaria*, *Pediococcus pentosaceus*, and *Lactiplantibacillus plantarum* were the main species. In addition, it was found that isolates of *Weissella cibaria* were mainly isolated in the early stage, which was consistent with the results. Besides, *Lactiplantibacillus plantarum* was mainly isolated in the middle and late stages of fermentation, and the *Pediococcus pentosaceus* isolates were present throughout the entire fermentation process, but were not the main species identified. Although the results of high-throughput sequencing showed that *Weissella hellenica*, *Weissella* sp., and *Latilactobacillus sakei* were dominant strains, the number of their isolates was also very small. Moreover, *Lactiplantibacillus pentosus* and *Lactiplantibacillus brevis* were not isolated and identified by culture-dependent methods, but these two species were found to be the main species in the end fermentation of PDM using high-throughput sequencing. These microorganisms were not recovered under the MRS culture conditions used in this study, possibly because the medium was not suitable for their growth. In future research, it is necessary to find a culture medium that is more suitable for the growth of *Latilactobacillus sakei*. Meanwhile, other LAB were also isolated, including *Latilactobacillus curvatus*, *Lactobacillus* sp., *Lactiplantibacillus plantarum*, *Loigolactobacillus coryniformis*, *Lactiplantibacillus paraplantarum*, and *Loigolactobacillus coryniformis* subsp. *torquens* and *Lactiplantibacillus argentoratensis*.

### 3.7. The Diversity Indices and Linear Discriminant Analysis (LDA) Effect Size (LEfSe) Analysis

**Figure 5:**
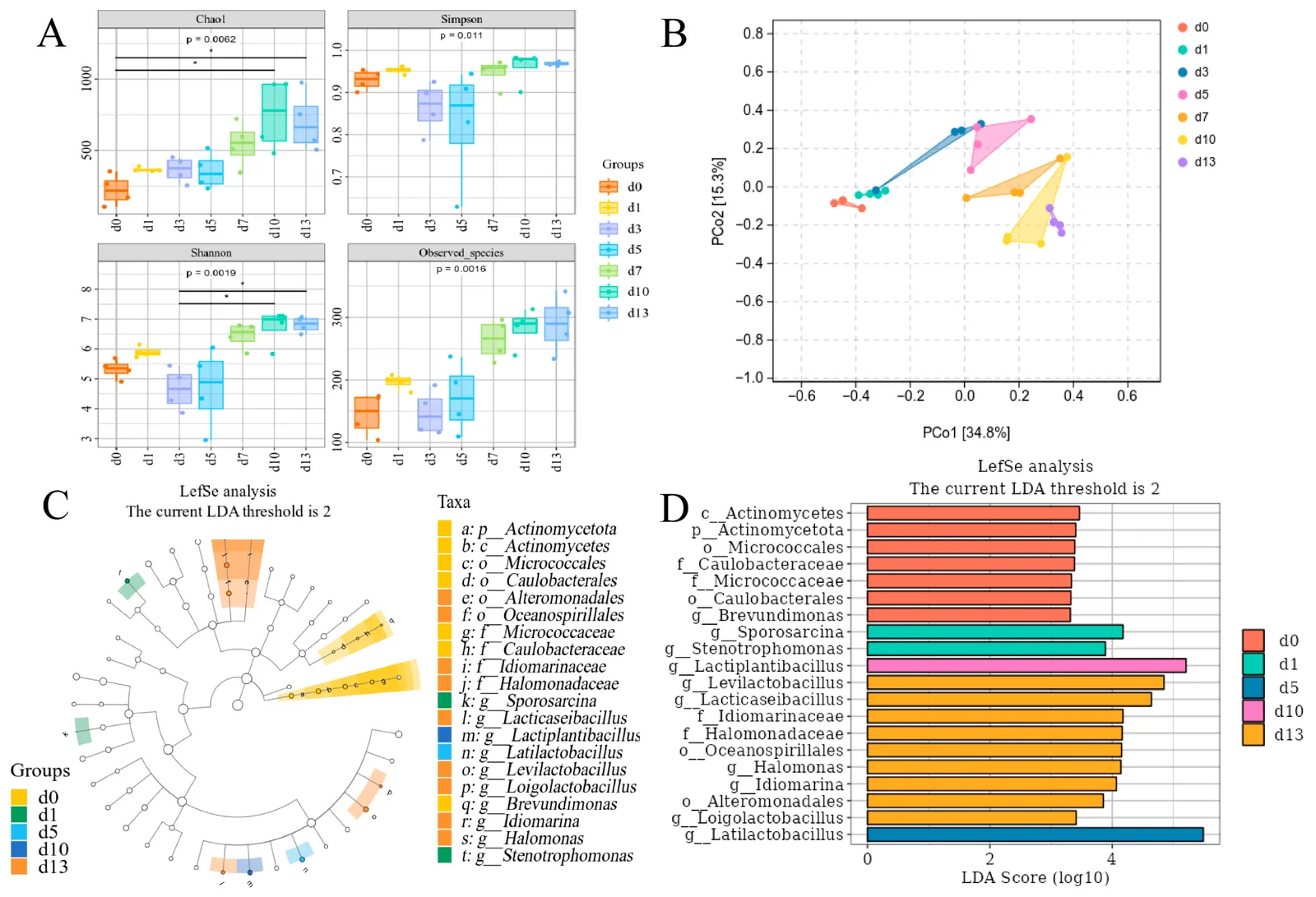
The bacterial alpha diversity, community structure, and biomarkers of PDM. (**A**) Alpha diversity indicies. (**B**) Principal coordinates analysis (PCoA). (**C**) The linear discriminant analysis effect size (LEfSe) analysis of phylogeny and abundance differences. (**D**) Linear discriminant analysis effect size (LDA). * *p* < 0.05.

The alpha-diversity indices of bacteria in the PDM fermentation increased with fermentation time (Figure 5A), while the alpha-diversity indices of fungi in the PDM fermentation decreased with fermentation time (Figure S2A). Principal coordinate analysis (PCoA) (Figure 5B and Figure S2B) revealed that the structures of both the bacterial and fungal communities in PDM changed significantly during fermentation.

LEfSe analysis (LDA > 3.5, *p* < 0.05; Figure 5C and Figure S2C) was used to identify biomarkers for PDM, identifying 20 different bacterial taxa (Figure 5D), including 13 on day 0, 2 on day 1, 1 on day 5, 1 on day 10, and 9 on day 13. *Lactiplantibacillus*, *Levilactobacillus*, *Lacticaseibacillus*, and *Latilactobacillus* were the main biomarker taxa. *Latilactobacillus* was significantly different from the others on day 5. *Sporosarcina* sp. was the biomarker on day 1, but *Latilactobacillus* sp. was the biomarker on day 5, and *Lactiplantibacillus* sp. was the biomarker on day 10. *Levilactobacillus* sp., *Lacticaseibacillus* sp., *Halomonas* sp., and *Idiomarina* sp. were most abundant on day 13. At the fungal genus level, *Filobasidium*, *Sporpbolomyces*, *Cryptococcus*, and *Kurtzmmaniella* differed significantly between these taxa (Figure S2D). On day 0, the *Basidionmycoda*, *Filobasidium*, *Filobasidiaceae*, *Filobasidiates*, *Sporpbolomyces*, *Cryptococcaceae*, *Bultera*, *Bulleraceae*, *Rhodotorula*, *Sporidiobolales*, *Sporidiobolaceae*, *and Microbotryomycetes* were the biomarkers in PDM. On day 1, *Kurtzmaniella* was the biomarker. Additionally, *Saccharomycetes*, *Ascomycota*, *and Saccharomycetales* were the biomarkers on day 7. These findings confirm that differences in processing methods and environmental conditions during natural fermentation lead to varying microbial metabolic rates, resulting in quality inconsistency.

### 3.8. Correlation Analysis of Microbiome Composition with Environmental Factors and Flavor Compound Composition During the Pickled and Dried Mustard Fermentation

**Figure 6:**
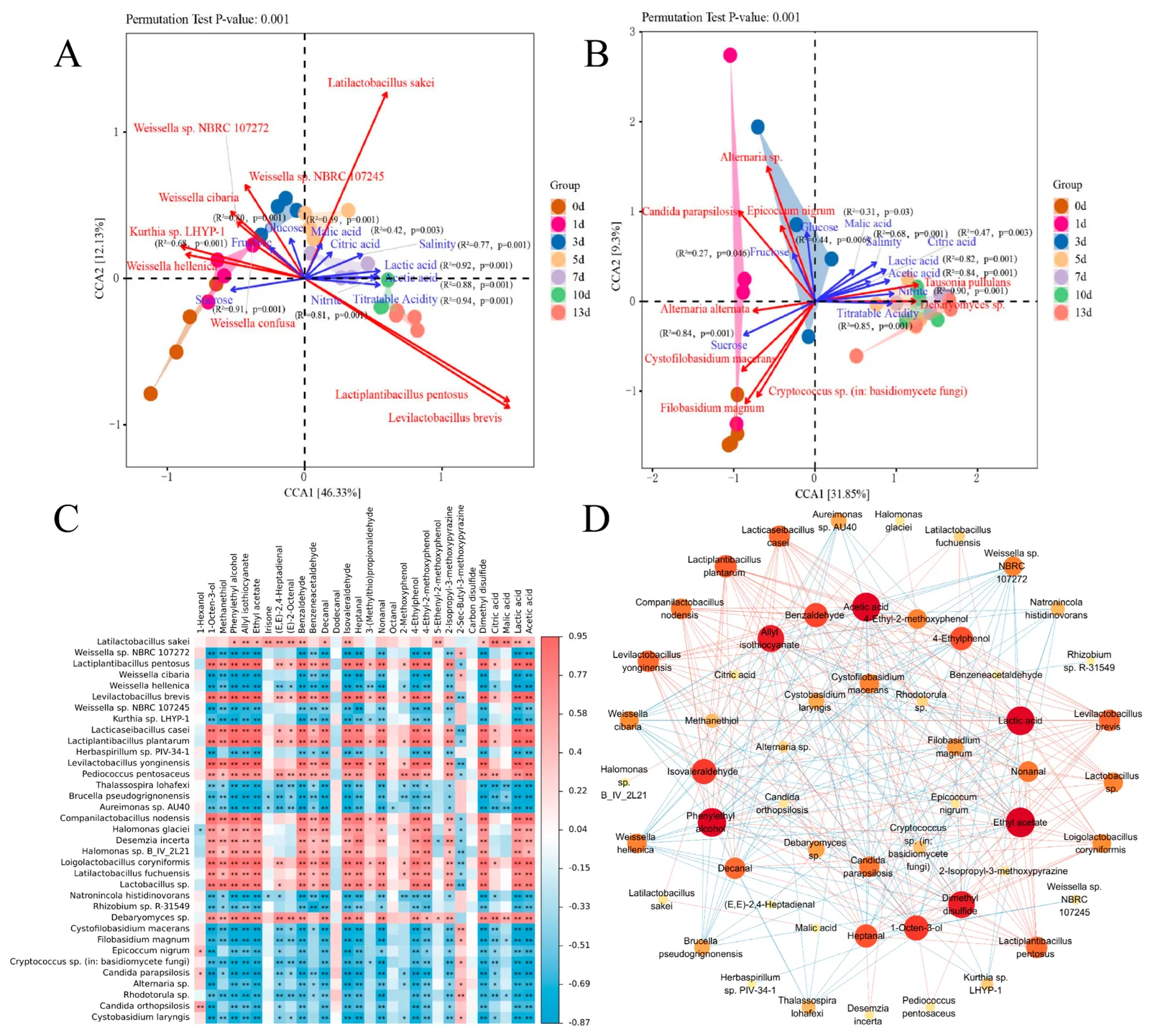
The correlation between microbiota, flavor, and environmental factors. Relationship between predominant (**A**) bacterial (the top 50 by relative abundance) and (**B**) fungal (the top 50 by relative abundance) species and physicochemical properties (pH, titratable acidity, salinity, nitrite, sucrose, glucose, and fructose) by CCA. Spearman correlation analysis between volatile flavor compounds and bacterial (top 50 relative abundance) and fungal (top 50 relative abundance) species, heatmap (**C**) and network diagram (**D**), ** *p* < 0.01, * *p* < 0.05.

Canonical correspondence analysis (CCA) was performed to explore the association of bacterial microbiota with environmental parameters in PDM. The total constrained variation was represented by CCA 1 (46.33%) and CCA 2 (12.13%) (Figure 6A). *Latilactobacillus sakei* showed a positive association with the glucose, salinity, malic acid, and citric acid contents. *Lactiplantibacillus pentosus* and *Levilactobacillus brevis* showed a positive association with the nitrite, lactic acid, and acetic acid contents, salinity, and titratable acidity, but showed a negative association with the glucose, fructose, and sucrose content. Many species in the genus *Weissella* and *Kurthia* sp. *LHYP-1* showed a positive association with the glucose, fructose, and sucrose content, but showed a negative association with the nitrite, lactic acid, and acetic acid contents, and titratable acidity. These findings were consistent with high-throughput sequencing results, showing that Weissella dominated the early stage of PDM fermentation, and *Latilactobacillus sakei*, *Lactiplantibacillus pentosus*, and *Levilactobacillus brevis* were the main microorganisms in the middle and end fermentation of PDM. Overall, lactic acid and acetic acid were the key factors driving microbial succession throughout PDM fermentation.

Fungal microbiome CCA revealed that the first two axes accounted for 31.85% (CCA 1) and 9.3% (CCA 2) of the constrained variation (Figure 6B). *Debaryomyces* sp. and *Tausonia pullulans* showed a positive association with the nitrite, salinity, lactic acid, acetic acid, citric acid, and malic acid contents, and titratable acidity. *Epicoccum nigrum*, *Candida parapsilosis*, and *Alternaria* sp. showed a positive association with the fructose and glucose content. *Filobasidium magnum*, *Cystofilobasidium macerans*, *Alternaria alternata*, and *Cryptococcus* sp. showed a positive association with the sucrose content, but showed a negative association with the nitrite, salinity, lactic acid, acetic acid, citric acid, and malic acid contents, and titratable acidity.

In short, whether it is bacteria or fungi in PDM, the results of CCA showed that their microbial species also change with the fermentation time, as reflected by the temporal changes in both bacterial and fungal species. Previous study revealed that the besides fermentation time, similar to other fermented vegetables [71,72], the microbial community in the PDM, such as *Weissella*, *Lactobacillus* and *Candida*, could also be affected by salt concentration and titratable acidity the microbial community of PDM. In addition to these environmental factors, temperature has also been reported to have an impact on fermented vegetable microorganisms [73]. Moreover, fermenting containers have been reported to affect the abundance of *Pichia* and *Candida* in Sichuan pickle [74]. Given that environmental factors strongly influence the microbial communities of fermented vegetables, future studies should investigate their specific effects on the PDM microbiome.

Spearman correlation analysis examined the relationship between 30 different flavor compounds (4 OAs and 26 VFCs) and 50 relatively abundant microbial taxa. Of these, 25 bacteria and 10 fungi strongly correlated with 30 flavor compounds (Figure 6C,D). In these main VFCs and OAs, 1-octen-3-ol, ethyl acetate, benzaldehyde, methanethiol, allyl isothiocyanate, phenylacetaldehyde, decanal, isovaleraldehyde, heptanal, nonanal, phenylethyl alcohol, 4-ethylphenol, 4-ethyl-2-methoxyphenol, 2-isopropyl-3-methoxypyrazine, dimethyl disulfide, lactic acid and acetic acid were positively correlated with *Latilactobacillus sakei*, *Lactiplantibacillus pentosus*, *Levilactobacillus brevis*, *Lacticaseibacillus casei*, *Lactiplantibacillus plantarum*, *Levilactobacillus yonginensis*, *Pediococcus pentosaceus*, *Companilactobacillus nodensis*, *Halomonas glaciei*, *Desemzia incerta*, *Halomonas* sp. *B_IV_2L21*, *Loigolactobacillus coryniformis*, *Latilactobacillus fuchuensis*, *Lactobacillus* sp., and *Debaryomyces* sp., but were negatively correlated with other microorganisms, such as *Weissella* sp. *NBRC 107272*, *Weissella cibaria*, *Weissella hellenica*, *Cystofilobasidium macerans*, and *Filobasidium magnum*, etc. Several of these microorganisms are known as starter cultures that can enhance flavor compound production. For example, the inoculation of *Lactiplantibacillus plantarum* and *Latilactobacillus sakei* has also been reported to improve the formation of 1-octen-3-ol, benzaldehyde, phenylacetaldehyde, heptanal, nonanal, and acetic acid [65]. The inoculation with *Lactiplantibacillus pentosus* has been reported to improve the formation of phenylethyl alcohol, 4-ethylphenol, and decanal [66], and the inoculation of *Levilactobacillus brevis* has been reported to improve the formation of ethyl acetate [67]. In total, most of these VFCs, such as nonanal, phenylethyl alcohol, dimethyl disulfide, 1-octen-3-ol, 4-ethylphenol, benzaldehyde, decanal, and heptanal, have also been reported to be related to *Lactobacillus* and *Pediococcus* (*Liquorilactobacillus nagelii*, *Pediococcus ethanolidurans*, *Lentilactobacillus buchneri*) in fermented vegetables, indicating that the formation of these main VFCs was closely related to microorganisms [8,56,75]. In addition, previous studies also showed that the abundance of *Weissella* decreased with prolonged fermentation and elevated lactic and acetic acids, indicating that *Weissella cibaria* mainly exists in the early and middle stages of fermented vegetables and its acid resistance is weak [18,76]. In total, although based on the relationship between high-throughput sequencing and flavor substances and previous relevant reports [65,66,67], many of the core microorganisms in this study have an important impact on the characteristic flavor; future studies should further verify the impact of core microorganisms on the fermentation of characteristic flavor compounds in PDM, aiming to promote its industrial production.

### 3.9. PICRUSt2 Prediction of Bacterial and Fungal Microbiome Functions and Potential Metabolic Network Construction

**Figure 7:**
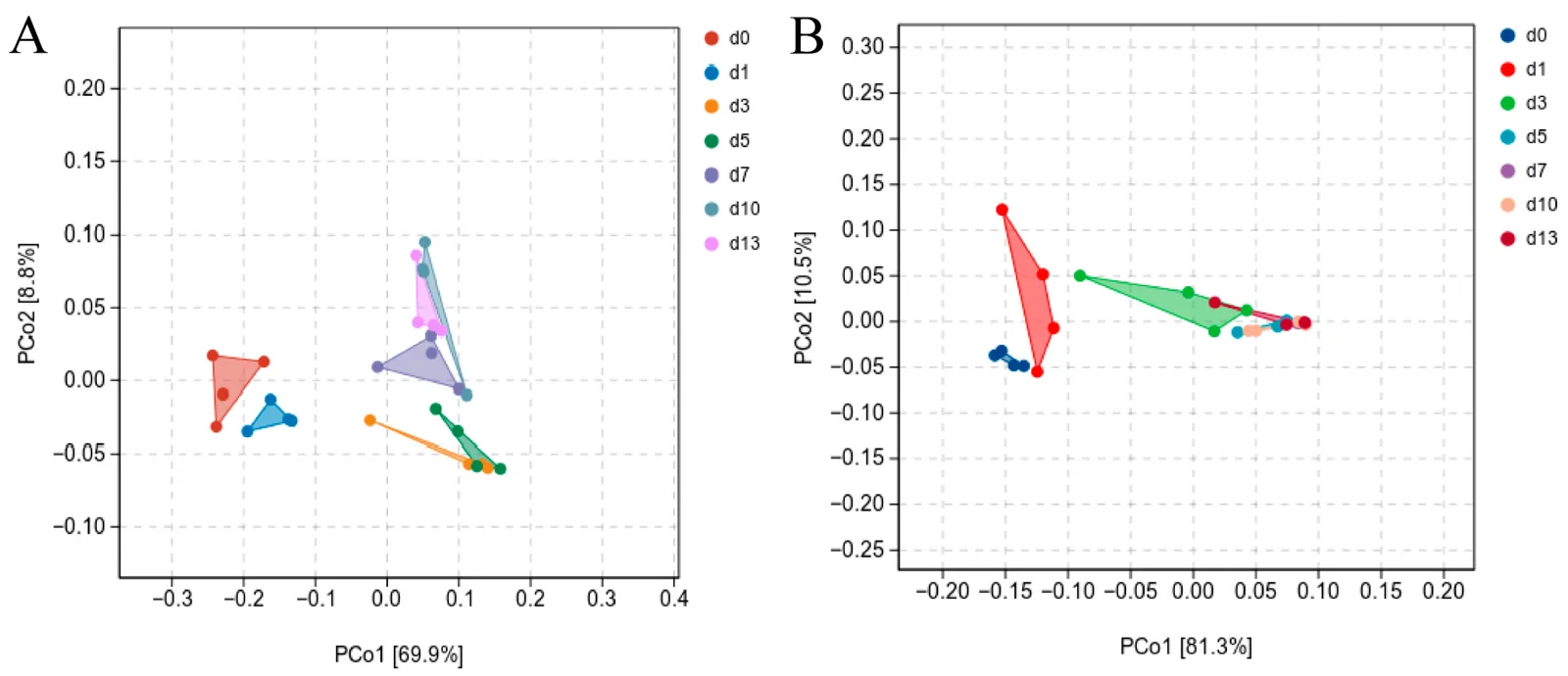
The principal coordinate analysis (PCoA) of metabolic function during PDM fermentation. (**A**) Bacteria. (**B**) Fungi.

Predictive functional profiles of the PDM bacterial and fungal microbiomes were generated using PICRUSt2, based on 16S rRNA and ITS rRNA sequencing datasets. PCoA analysis revealed that the functional structure changed as fermentation proceeded (Figure 7A,B). The predicted functions of the bacterial microbial flora were classified into six categories: metabolism, genetic information processing, environmental information processing, cellular processes, human diseases, and organismal systems. The *metabolism* function was the major pathway in PDM (Figure S3A), as it is in dairy and vegetable products [77]. Carbohydrate metabolism, the most abundant pathway at KEGG level 2 (Figure S3B), serves as the primary energy source for bacteria in fermented foods. Under microbial and enzymatic catalysis, raw sugars and proteins are degraded to small-molecule metabolites, which are subsequently converted into secondary metabolites, including aroma-active compounds [77,78]. Among the KEGG functional class three pathways at the level of *carbohydrate metabolism*, the *pentose phosphate pathway* had the highest percentage (Figure S3C), whereas among the KEGG functional class three pathways at the level of *metabolism of cofactors and vitamins*, *thiamine metabolism* had the highest percentage (Figure S3D). The fungal functions in PDM were predicted and classified into five groups: *biosynthesis*, *degradation/utilization/assimilation*, *precursor metabolite and energy production*, *glycan pathways*, and *metabolism clusters*. Among them, biosynthesis exhibited the highest relative content (44.09%) and constituted the major functional activity of the microbial flora during PDM fermentation (Figure S4A). At the KEGG secondary classification level, *nucleoside and nucleotide biosynthesis* had the highest relative abundance and was the major pathway (Figure S4B). At the level three KEGG functional class pathway of *nucleoside and nucleotide biosynthesis*, *pyrine nucleotide biosynthesis* had the highest percentage (Figure S4C), whereas at the level of *cofactor*, *prosthetic group*, *electron carrier*, and *vitamin biosynthesis*, *vitamin biosynthesis* had the highest percentage of the three-level KEGG functional class pathways (Figure S4D). However, PICRUSt prediction is dependent on databases and mathematical methods, which cannot reflect all the functional genes involved in metabolic pathways in a complete and precise manner. Therefore, further studies will be needed to obtain a more thorough understanding.

**Figure 8:**
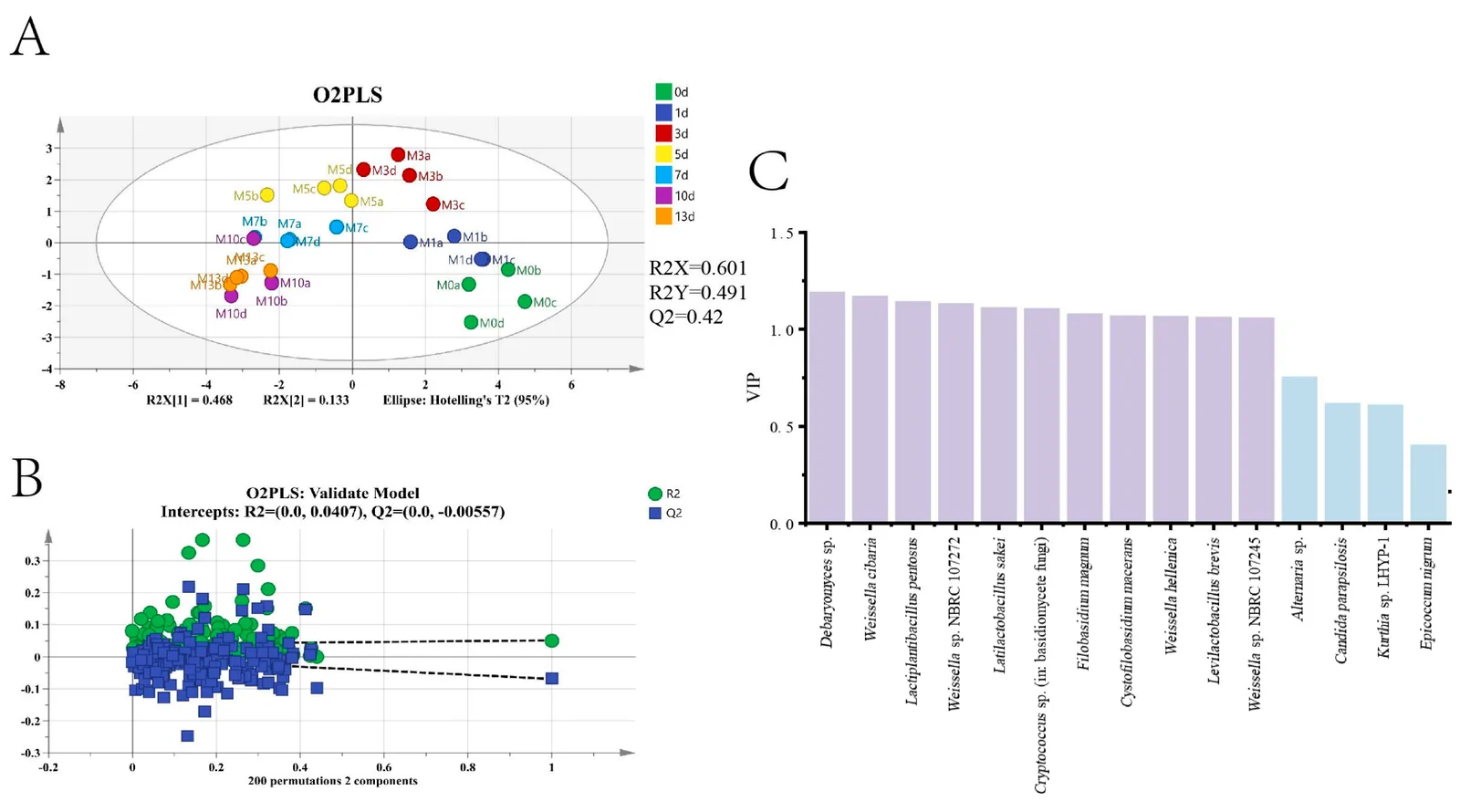
Correlation between microbial species and flavor metabolites during PDM fermentation based on the O2PLS model. (**A**) O2PLS score plot. (**B**) Validation plot of the O2PLS model. (**C**) Predictive variable importance for the projection (VIP) plot.

Based on previous studies [79], we selected core flavor-related microbes based on three rules: they must be stable during fermentation, strongly correlate with at least one flavor compound (|r| > 0.7), and have a VIP > 1.0 in the O2PLS model. The results showed that the model exhibited good and stable performance (Figure 8A,B). Combined with the Spearman correlation analysis (|r| > 0.7, *p* < 0.05) and the stability of these species in the PDM fermentation process, we have identified a total of eleven species with VIP > 1.0 that were candidate core microorganisms potentially related to the flavor of PDM, including *Debaryomyces* sp., *Weissella cibaria*, *Lactiplantibacillus pentosus*, *Weissella* sp. *NBRC 107272*, *Latilactobacillus sakei*, *Cryptococcus* sp., *Filobasidium magnum*, *Cystofilobasidium macerans*, *Weissella hellenica*, *Levilactobacillus brevis*, and *Weissella* sp. *NBRC 107245* (Figure 8C) were the core microbes during the PDM fermentation. In other reports of fermented vegetables, *Weissella* and *Lactobacillus* are also reported to be the core microbes, contributing to their characteristic flavors [50,64]. *Weissella cibaria* contributed to the production of esters (e.g., ethyl acetate), aldehydes (e.g., benzaldehyde), and alcohols (e.g., 1-octen-3-ol) through heterolactic fermentation [80]. *Latilactobacillus sakei* and *Lactiplantibacillus pentosus* were linked to the accumulation of nonanal, heptanal, phenylacetaldehyde, phenylethyl alcohol, 4-ethylphenol, and decanal [81]. Likewise, Debaryomyces, which was a core microbe in our study, has been found in Sichuan radish paocai and northeastern sauerkraut [69,70], and could produce many key enzymes for flavor formation of fermented vegetables. *Debaryomyces hansenii*, a dominant fungus in the middle and late stages, produced decarboxylase and vinylphenol reductase that convert *p*-coumaric acid and ferulic acid to 4-ethylphenol and 4-ethylguaiacol, and also contributed to the biosynthesis of methylthio alcohols [82]. Future research could utilize these candidate core microbial species as indicators of community structure to explore the mechanisms of fermentation and flavor generation in PDM.

**Figure 9:**
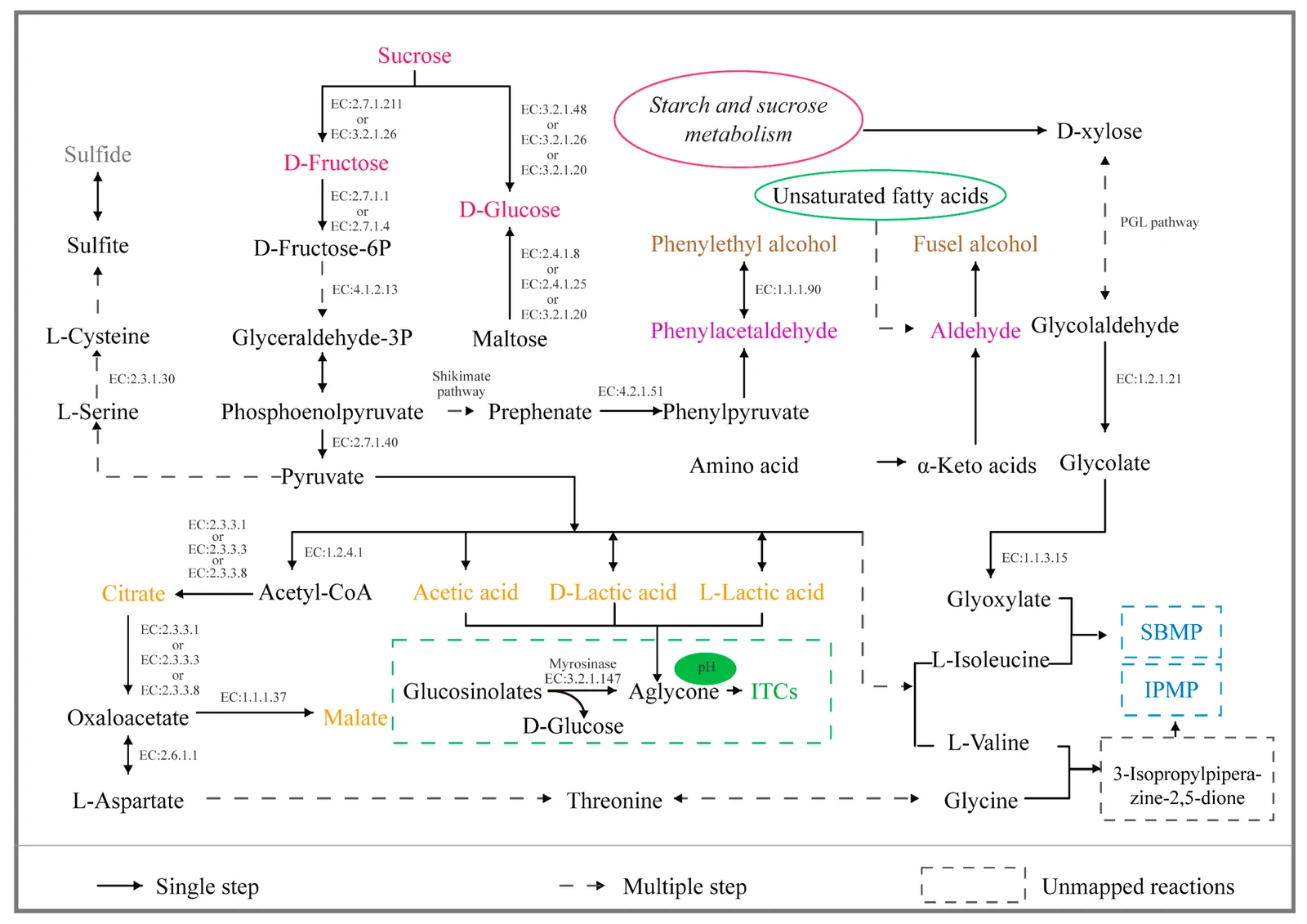
The reconstruction of the simplified metabolic pathway of specified flavor compounds and their precursors.

From the predicted metabolic functions of the PDM microbiota, simplified pathways for key flavor compounds and their precursors were derived (Figure 9), along with enzyme assignments to each pathway (Table S7). Carbohydrates are metabolized via the glycolysis pathway to produce important intermediate metabolites such as glyceraldehyde-3P, phosphoenolpyruvate, pyruvate, and acetyl-CoA. Pyruvate can then be converted to lactic, acetic, citric, and malic acids via pyruvate metabolism and the TCA cycle. Glyceraldehyde-3P is converted to cysteine, which is further metabolized to sulfites and sulfides via sulfur, cysteine, and methionine metabolism. There are various higher alcohols in the PDM flavor profile, which can be formed to produce α-keto acids by aminotransferase, then decarboxylated by decarboxylases to produce aldehydes, and finally reduced to alcohols by alcohol dehydrogenase [83,84]. Most long-chain aldehydes are produced by the oxidation of unsaturated fatty acids such as linoleic acid, linolenic acid, and arachidonic acid [79]. Glucosinolates can be converted by myrosinase into glycosides, which are further degraded to isothiocyanates [85]. Lactic and acetic acids produced during PDM fermentation regulate isothiocyanate production by altering the environmental pH [62]. 3-Isopropylpiperazine-2,5-dione produced by the condensation of valine and glycine can undergo a series of reactions to produce isopropyl methylphenol [86]. L-isoleucine is amidated and condensed with glyoxalate to produce 2-*sec*-butyl-3-methoxypyrazine via a methylation reaction [87]. Accordingly, a potential approach to control PDM flavor is provided through targeted microbial manipulation during fermentation. Although we have reconstructed its potential metabolic network, further validation and reshaping through metatranscriptomics and metabolomics are needed in our future research.

## 4. Conclusions

The physicochemical properties, microbiome composition, and contents of OAs, reducing sugars, amino acids, and VFCs in the PDM fermentation were studied. During fermentation, the main aromas of PDM are similar to pine and orange oils. As the fermentation progressed, the phenolic and aldehyde contents increased significantly; the major aldehydes in PDM were dodecanal, nonanal, and phenylacetaldehyde. The change in microbial abundance during PDM fermentation was a gradual transition from *Weissella*, *Basidiomycota* to *Latilactobacillus*, and *Ascomycota*, respectively. The candidate core microorganisms potentially related to PDM flavor formation included *Debaryomyces* sp., *Weissella cibaria*, *Lactiplantibacillus pentosus*, and *Weissella* sp. *NBRC 107272*, *Latilactobacillus sakei*, *Cryptococcus* sp., *Filobasidium magnum*, *Cystofilobasidium macerans*, *Weissella hellenica*, *Levilactobacillus brevis*, and *Weissella* sp. *NBRC 107245*. They are strongly correlated with many important flavor metabolites produced during the fermentation of PDM. Changes in the microbiome composition and flavor profile of PDM as the fermentation progressed were elucidated, providing a deeper understanding of the physicochemical characteristics and candidate core bacteria and fungi associated with the PDM fermentation. For example, *Latilactobacillus sakei* and *Lactiplantibacillus pentosus* were positively correlated with nonanal, heptanal, phenylacetaldehyde, phenylethyl alcohol, 4-ethylphenol, decanal, lactic acid, and acetic acid, whereas *Weissella cibaria* and *Weissella hellenica* were negatively correlated with these compounds. In addition, *Debaryomyces* sp. was positively correlated with 1-octen-3-ol, ethyl acetate, and benzaldehyde. The improved understanding of the volatile flavor compound profile will facilitate future process and quality control to maintain and enhance the unique flavor of PDM. The improved understanding of the relationship between the microbial composition of the fermentation, the flavor compound profile, and the metabolic pathways that produce the flavor compounds will also improve the quality control of the fermentation process and facilitate future industrialization. However, the detailed biosynthetic mechanisms of the flavor compounds and their relationships with the PDM microbiome still need clarification by metatranscriptomics. Future research should apply metatranscriptomics to obtain a more detailed understanding of the specific microorganisms associated with the characteristic PDM flavor compounds and use them to improve the quality control and industrialization of PDM production.

## Data Availability

The original contributions presented in this study are included in the article/Supplementary Materials. Further inquiries can be directed to the corresponding author.

## Supplementary Materials

The following supporting information can be downloaded at: https://www.mdpi.com/article/10.3390/foods15193486/s1, Figure S1: Phylogenetic tree based on the 16S rDNA sequences constructed using the neighborhood-joining method; Figure S2: The fungal alpha diversity, community structure, and Biomarkers of PDM. (A) Alpha diversity index. (B) Principal coordinates analysis (PCoA). (C) The linear discriminant analysis effect size (LEfSe) analysis of phylogeny and abundance differences. (D) Linear discriminant analysis effect size (LDA). *p* < 0.001, * *p* < 0.05; Figure S3: The metabolism functions of bacterial communities in PDM during fermentation. (A) The relative abundance of KEGG pathways. (B) The relative abundance of pathways in metabolism. (C) The relative abundance of pathways in carbohydrate metabolism. (D) The relative abundance of pathways in the metabolism of cofactors and vitamins; Figure S4: The biosynthesis functions of fungal communities in PDM during fermentation. (A) The relative abundance of KEGG pathways. (B) The relative abundance of pathways in biosynthesis. (C) The relative abundance of pathways in nucleoside and nucleotide biosynthesis. (D) The relative abundance of pathways in cofactor, prosthetic group, electron carrier, and vitamin biosynthesis; Table S1: The taste activity value of free amino acids during the pickled and dried mustard fermentation; Table S2: The taste activity value of free amino acids during the pickled and dried mustard fermentation; Table S3: The detail identification information of all volatile flavor compounds; Table S4: 26 Aromatic active compounds; Table S5: Number of isolates from the PDM samples summarized at the species level for LAB by using culture-dependent methods; Table S6: The closest related type strains of sequences of isolates from PDM in NCBI Genbank; Table S7: The annotation of enzymes in simplified metabolic pathways of key flavor compounds and their precursors.
