# Supplementary material for: Exploring Dynamic Changes in Microbial Succession and Flavor-Active Metabolites During Pickled and Dried Mustard Fermentation of Traditional Fermented Vegetables
Source: Foods. 2026 Sep 29;15(19):3486. doi: 10.3390/foods15193486 (PMC13665255; doi:10.3390/foods15193486)
Supplement: Supplementary file 1 [file foods-15-03486-s001.zip › foods-4539483-supplementary.pdf]

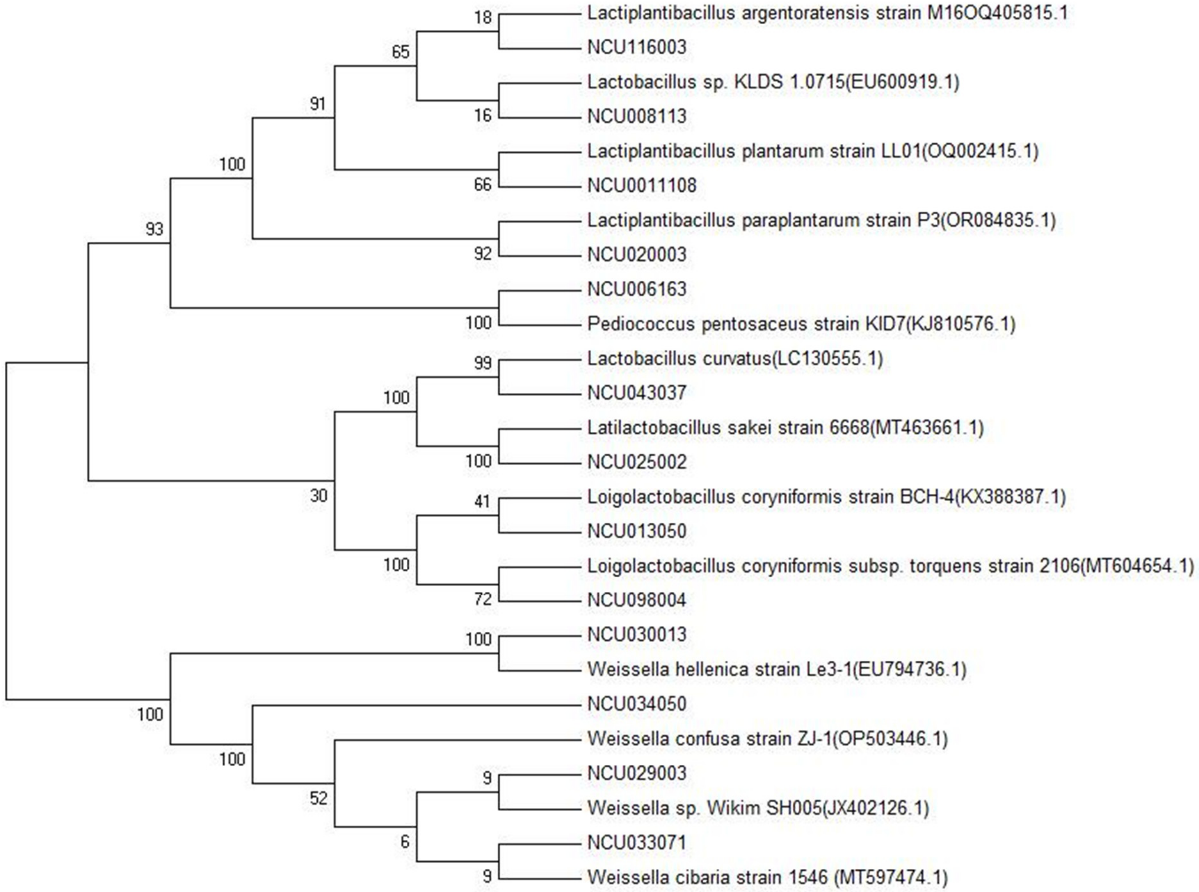

Figure S1. Phylogenetic tree based on the 16S rDNA sequences constructed using the neighborhood-joining method.

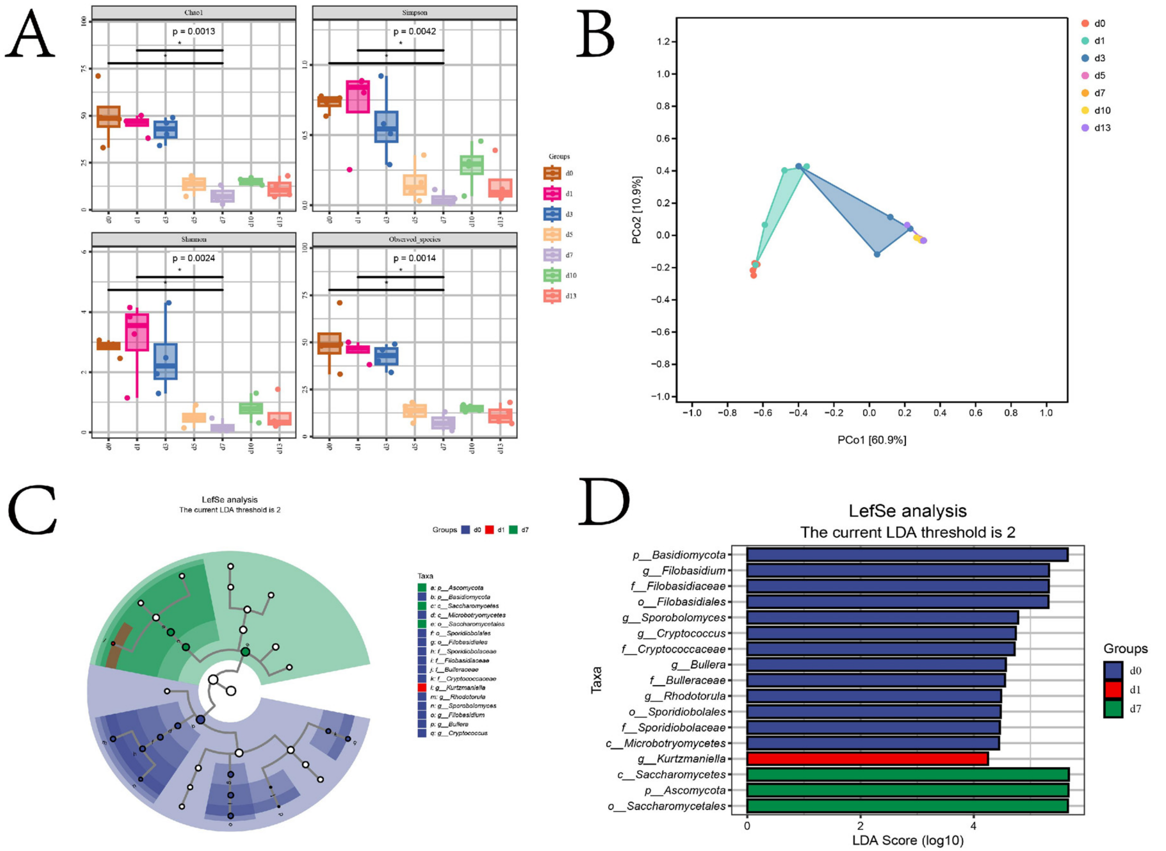

Figure S2. The fungal alpha diversity, community structure, and Biomarkers of PDM. (A) Alpha diversity index. (B) Principal coordinates analysis (PCoA). (C) The linear discriminant analysis effect size (LEfSe) analysis of phylogeny and abundance differences. (D) Linear discriminant analysis effect size (LDA). P < 0.001, *P < 0.05.

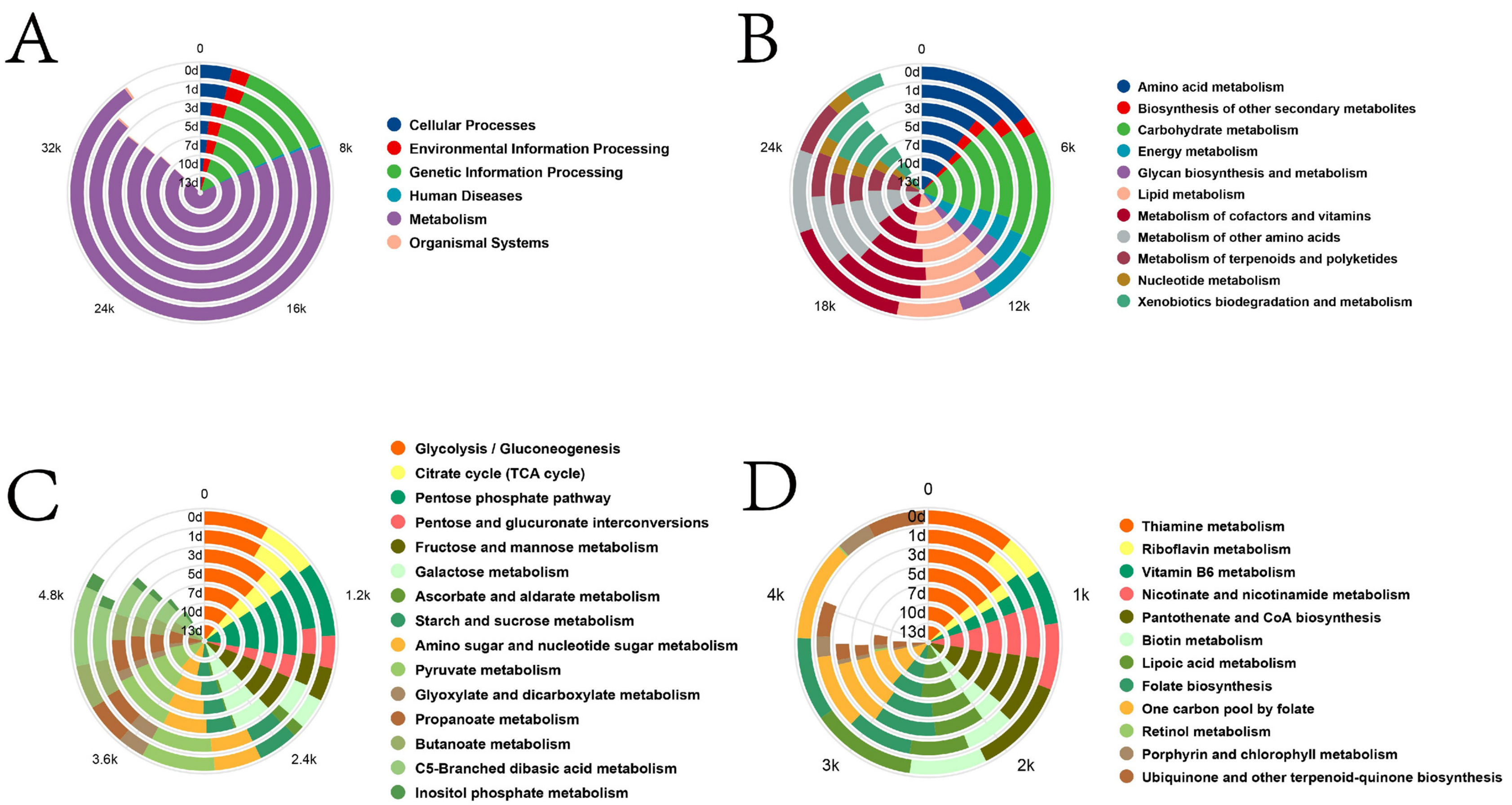

Figure S3. The metabolism functions of bacterial communities in PDM during fermentation. (A) The relative abundance of KEGG pathways. (B) The relative abundance of pathways in metabolism. (C) The relative abundance of pathways in carbohydrate metabolism. (D) The relative abundance of pathways in the metabolism of cofactors and vitamins.

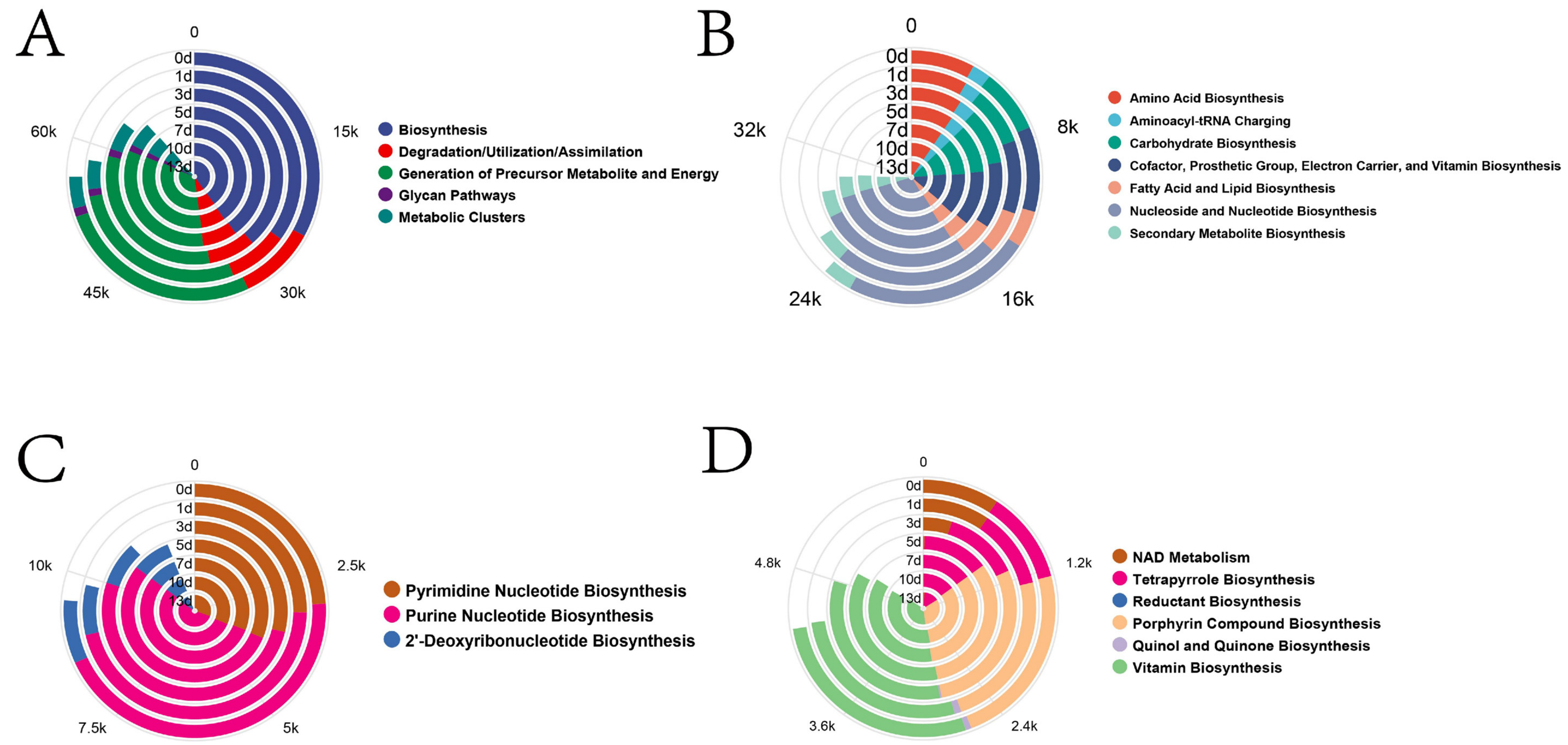

Figure S4. The biosynthesis functions of fungal communities in PDM during fermentation. (A) The relative abundance of KEGG pathways. (B) The relative abundance of pathways in biosynthesis. (C) The relative abundance of pathways in nucleoside and nucleotide biosynthesis. (D) The relative abundance of pathways in cofactor, prosthetic group, electron carrier, and vitamin biosynthesis.

Table S1 The taste activity value of free amino acids during the pickled and dried mustard fermentation.

Organic acid	Taste	Thresholds[a] (g/L)	TAV[b]						
			0d	1d	3d	5d	7d	10d	13d
Citric acid	Sour	0.499	0.38	0.56	1.09	2.14	2.10	1.10	1.00
Malic acid	Sour	0.496	0.53	0.84	1.68	2.44	1.76	1.27	1.04
Lactic acid	Sour	1.261	0.00	1.78	3.46	4.08	4.37	5.09	5.81
Acetic acid	Sour	0.120	0.00	8.49	16.70	18.61	29.99	35.96	31.10

a The threshold of each compound was obtained from [1].

b TAV, taste active value.

Table S2 The taste activity value of free amino acids during the pickled and dried mustard fermentation.

Amino acid	Taste	Thresholds[a] (mg/g)	TAV[b]						
			0d	1d	3d	5d	7d	10d	13d
Asp	Umami	1	0.00	0.00	0.00	0.00	0.00	0.00	0.00
Glu	Umami	0.3	0.02	0.02	0.03	0.03	0.03	0.03	0.02
Thr	Sweet	2.6	0.00	0.00	0.00	0.00	0.00	0.00	0.00
Ser	Sweet	1.5	0.01	0.01	0.01	0.01	0.01	0.01	0.00
Gly	Sweet	1.3	0.00	0.00	0.00	0.00	0.00	0.00	0.00
Ala	Sweet	0.6	0.00	0.01	0.01	0.01	0.01	0.01	0.02
Lys	Sweet	0.5	3.20	4.46	3.98	4.06	2.92	1.14	0.78
Arg	Bitter	0.5	0.01	0.01	0.01	0.01	0.00	0.00	0.00
Val	Bitter	0.4	0.01	0.01	0.01	0.01	0.01	0.01	0.01
Met	Bitter	0.3	0.00	0.00	0.00	0.00	0.00	0.00	0.00
Ile	Bitter	0.9	0.00	0.00	0.00	0.00	0.00	0.00	0.00
Leu	Bitter	1.9	0.00	0.00	0.00	0.00	0.00	0.00	0.00
Tyr	Bitter	0.9	0.00	0.00	0.00	0.00	0.00	0.00	0.00
Phe	Bitter	0.2	0.01	0.02	0.02	0.02	0.02	0.02	0.02
His	Bitter	0.9	0.00	0.00	0.00	0.00	0.00	0.00	0.00
Cys	Tasteless	/	/	/	/	/	/	/	/

/, not available.

a Threshold of each component taken from the following literature [2].

b TAV, taste active value.

Table S3 The detail identification information of all volatile flavor compounds.

Compounds	Formula	CAS Number	RI
2-Propenal	C_3H_4O	107-02-8	810
2-Methyl-propanal	C_4H_8O	78-84-2	817
Methional	C_4H_8OS	3268-49-3	1461
3-Methyl-butanal	$C_5H_{10}O$	590-86-3	938
Pentanal	$C_5H_{10}O$	110-62-3	968
2-Hexenal	$C_6H_{10}O$	505-57-7	1187
Hexanal	$C_6H_{12}O$	66-25-1	1058
(E,E)-2,4-Heptadienal	$C_7H_{10}O$	4313-03-5	1524
(E)-2-Heptenal	$C_7H_{12}O$	18829-55-5	1357
Heptanal	$C_7H_{14}O$	111-71-7	1152
Benzaldehyde	C_7H_6O	100-52-7	1506
(E)-2-Octenal	$C_8H_{14}O$	2548-87-0	1468
Octanal	$C_8H_{16}O$	124-13-0	1302
Phenylacetaldehyde	C_8H_8O	122-78-1	1644
(E,Z)-2,6-Nonadienal	$C_9H_{14}O$	557-48-2	1534
(E)-2-Nonenal	$C_9H_{16}O$	18829-56-6	1535
Nonanal	$C_9H_{18}O$	124-19-6	1402
Decanal	$C_{10}H_{20}O$	112-31-2	1521
Dodecanal	$C_{12}H_{24}O$	112-54-9	1739
(Z)-2-Penten-1-ol	$C_5H_{10}O$	1576-95-0	1356
1-Penten-3-ol	$C_5H_{10}O$	616-25-1	1146
Cyclopentanol	$C_5H_{10}O$	96-41-3	1349
2-Methyl-1-butanol	$C_5H_{12}O$	137-32-6	1238
(E)-3-Hexen-1-ol	$C_6H_{12}O$	928-97-2	1347
Cyclohexanol	$C_6H_{12}O$	108-93-0	1437
1-Hexanol	$C_6H_{14}O$	111-27-3	1390
Phenylethyl alcohol	$C_8H_{10}O$	60-12-8	1924
Methylbenzylalcohol	$C_8H_{10}O$	98-85-1	1827
1-Octen-3-ol	$C_8H_{16}O$	3391-86-4	1412
2-Ethyl-1-hexanol	$C_8H_{18}O$	104-76-7	1504
Acetone	C_3H_6O	67-64-1	827
2-Butanone	C_4H_8O	78-93-3	921
Acetoin	$C_4H_8O_2$	513-86-0	952
3-Pentanone	$C_5H_{10}O$	96-22-0	951
1-Penten-3-one	C_5H_8O	1629-58-9	1012
2,3-Pentanedione	$C_5H_8O_2$	600-14-6	1024
3-Methyl-2-pentanone	$C_6H_{12}O$	565-61-7	1017
6-Methyl-5-hepten-2-one	$C_8H_{14}O$	110-93-0	1321
Irisone	$C_{13}H_{20}O$	14901-07-6	1974

(E)-6,10-Dimethyl-5,9-undecadien-2-one	$C_{13}H_{22}O$	3796-70-1	1875
Methyl acetate	$C_3H_6O_2$	79-20-9	857
Ethyl acetate	$C_4H_8O_2$	141-78-6	857
Ethyl phenylacetate	$C_{10}H_{12}O_2$	101-97-3	1783
Phenylethyl acetate	$C_{10}H_{12}O_2$	103-45-7	1832
5-Hexyldihydro-2(3H)-furanone	$C_{10}H_{18}O_2$	706-14-9	2178
Ethyl caprylate	$C_{10}H_{20}O_2$	106-32-1	1428
2-Nitro-phenol	$C_6H_5NO_3$	88-75-5	1818
2-Methyl-phenol	C_7H_8O	95-48-7	2017
2-Methoxy-phenol	$C_7H_8O_2$	90-05-1	1871
4-Ethyl-phenol	$C_8H_{10}O$	123-07-9	2172
2-Methoxy-5-vinylphenol	$C_9H_{10}O_2$	621-58-9	1308
4-Ethyl-2-methoxy-phenol	$C_9H_{12}O_2$	2785-89-9	2012
Carbon disulfide	CS_2	75-15-0	786
Methanethiol	CH_4S	74-93-1	667
Dimethyl disulfide	$C_2H_6S_2$	624-92-0	1042
Diallyl disulphide	$C_6H_{10}S_2$	2179-57-9	1482
Dimethyl trisulfide	$C_{12}H_{24}O$	3658-80-8	1407
Benzene	C_6H_6	71-43-2	908
Styrene	C_8H_8	100-42-5	1228
Indole	C_8H_7N	120-72-9	2424
Ethylbenzene	C_8H_{10}	100-41-4	1160
1,3-Dimethyl-benzene	C_8H_{10}	108-38-3	1168
2-Isopropyl-3-methoxypyrazine	$C_8H_{12}N_2O$	25773-40-4	1422
2-*Sec*-butyl-3-methoxypyrazine	$C_9H_{14}N_2O$	24168-70-5	1507
2-Pentyl-furan	$C_9H_{14}O$	3777-69-3	1207
Acetic acid	$C_2H_4O_2$	64-19-7	1434
Pentanoic acid	$C_5H_{10}O_2$	109-52-4	1715
2-Methyl-butanoic acid	$C_5H_{10}O_2$	116-53-0	1689
Methane isothiocyanate	C_2H_3NS	556-61-6	1246
Allyl isothiocyanate	C_4H_5NS	57-06-7	1348
Dimethylamine	C_2H_7N	124-40-3	670
Sulfur dioxide	O_2S	7446-09-5	873
Anethole	$C_{10}H_{12}O$	104-46-1	1823
Hexadecane	$C_{16}H_{34}$	544-76-3	1612

RI: retention index (The RI of unknown compounds on the DB-Wax column calculated against the GC-MS retention time of n-alkanes (C5-C40).

Table S4 26 Aromatic active compounds

Aromatic active compounds	End of fermentation ROAV value	Flavor description
Dodecanal	100.00	soapy, waxy, aldehydic, citrus, orange, rind, floral
2-Isopropyl-3-methoxypyrazine	98.92	pea, green pea, earthy, beany, chocolate, nutty
2-*Sec*-butyl-3-methoxypyrazine	80.92	musty, pea, green pea, galbanum, pepper, bell pepper
Nonanal	60.26	waxy, aldehydic, citrus, fresh, green, lemon peel, cucumber, fatty
Phenylacetaldehyde	56.13	green, floral, rose, clover
Dimethyl disulfide	52.25	sulfurous cabbage, malt, cream
Methanethiol	23.72	garlic, cheese, onions, cabbage, eggs
Heptanal	17.00	green, aldehydic, oily, cortex, grassy, clover, cilantro
Carbon disulfide	12.91	rotten radish
Decanal	12.10	sweet, citrus, wax, floral
Allyl isothiocyanate	11.34	mustard seeds, irritant, foul odor, spicy taste
4-Ethylphenol	8.89	mold, meat, hyacinth, gardenia
Irisone	4.26	wood, sweet, berry
1-Octen-3-ol	4.17	mushroom, sweet, herb, hay, lavender, rose
Isovaleraldehyde	3.99	apple, citrus, lemon
3-(Methylthio)propionaldehyde	2.99	onion, meat, baked potatoes
5-Ethenyl-2-methoxyphenol	2.89	vanilla, sweet, smoky, woody, spicy
4-Ethyl-2-methoxyphenol	2.71	smoked, woody, spicy, slightly sweet, cloves herbs
(E)-2-octenal	2.55	cucumber, herbs, banana leaves, fat
Phenylethyl alcohol	2.19	sweet, rose
(E,E)-2,4-heptadienal	2.07	fried, nuts, grass, citrus
1-Hexanol	1.74	green, alcoholic, sweet, and light fats
Octanal	1.53	aldehydes, tropical fruits, ripe bananas, peat, smoked, coconut meat, cream, sweet oranges, lemon peel oil
Ethyl acetate	1.50	sweet, fruity, pear, apple
2-Methoxy-phenol	1.45	sweet, smoky
Benzaldehyde	1.13	bitter almonds, cherries, nuts

Table S5 Number of isolates from the PDM samples summarized at the species level for LAB by using culture-dependent methods

Species	Fermentation Time							Total
	0d	1d	3d	5d	7d	10d	13d	
Weissella cibaria	10	8	7	0	0	0	0	25
Pediococcus pentosaceus	3	3	1	6	5	4	1	23
Weissella confusa	1	0	0	0	0	0	0	1
Weissella sp	0	1	1	0	0	0	0	2
Latilactobacillus curvatus	0	1	1	0	0	0	0	2
Weissella hellenica	0	0	3	0	0	0	0	3
Lactiplantibacillus plantarum	0	0	0	5	5	10	8	28
Loigolactobacillus coryniformis	0	0	0	1	1	0	0	2
Latilactobacillus sakei	0	0	0	0	0	2	0	2
Loigolactobacillus coryniformis subsp. torquens	0	0	0	0	1	0	0	1
Lactobacillus sp	0	0	0	0	0	1	0	1
Lactiplantibacillus paraplantarum	0	0	0	0	0	0	1	1
Lactiplantibacillus argentoratensis	0	0	0	0	0	0	1	1
Total	14	13	13	12	12	17	11	92
Number of species detected	3	4	5	3	4	4	3	13

Table S6 The closest related type strains of sequences of isolates from PDM in NCBI Genbank

Isolates	Closest relative sequence	Accession number[a]	Length (bp)	Similarity (%)
NCU034050	*Weissella cibaria strain 1546*	MT597474.1	1485	100
NCU006163	Pediococcus pentosaceus strain KID7	KJ810576.1	1508	99.86
NCU033071	*Weissella confusa strain ZJ-1*	OP503446.1	1480	100
NCU025002	*Lactobacillus sakei strain 6668*	MT463661.1	1484	99.66
NCU029003	*Weissella sp. Wikim SH005*	JX402126.1	1488	99.93
NCU043037	*Lactobacillus curvatus*	LC130555.1	1454	99.86
NCU030013	*Weissella hellenica strain Le3-1*	EU794736.1	1482	99.86
NCU0011108	*Lactiplantibacillus plantarum strain LL01*	OQ002415.1	1472	99.86
NCU008113	*Lactobacillus sp. KLDS 1.0715*	EU600919.1	1475	99.86
NCU020003	*Lactiplantibacillus paraplantarum strain P3*	OR084835.1	1467	99.73
NCU116003	*Lactiplantibacillus argentoratensis strain M16*	OQ405815.1	1460	100.00
NCU013050	*Lactobacillus coryniformis strain BCH-4*	KX388387.1	1510	99.80
NCU098004	Lactobacillus coryniformis subsp. torquens strain 2106	MT604654.1	1476	99.80

[a] Accession number of the sequence of the closest relative found by BLAST search

Table S7 The annotation of enzymes in simplified metabolic pathways of key flavor compounds and their precursors.

Function	Enzyme name	EC number
Sucrose degradation	sucrose PTS system	EC:2.7.1.211
	alpha-glucosidase	EC:3.2.1.20
	beta-fructofuranosidas	EC:3.2.1.26
Gluconeogenesis	4-alpha-glucanotransferase	EC:2.4.1.25
	glycogen debranching enzyme	EC:2.4.1.25
	maltose phosphorylase	EC:2.4.1.8
	sucrose PTS system	EC:2.7.1.205
	6-phospho-beta-glucosidase	EC:3.2.1.86
	beta-glucosidase	EC:3.2.1.21
	alpha-glucosidase	EC:3.2.1.20
	maltose phosphorylase	EC:2.4.1.8
Glucose utilization	sucrose PTS system	EC 2.7.1.199
	aldose 1-epimerase	EC 5.1.3.3
	glucokinase	EC 2.7.1.2
	quinoprotein glucose dehydrogenase	EC 1.1.5.2
	glucose 1-dehydrogenase	EC 1.1.1.47
Lactic acid biosynthesis	NADPH-dependent methylglyoxal reductase	EC:1.1.1.283
	UDPglucose 6-dehydrogenase	EC:1.1.1.22
	D-lactate dehydrogenase	EC:1.1.1.28
	D-lactate dehydrogenase (quinone)	EC:1.1.5.12
	D-lactate dehydrogenase (cytochrome)	EC:1.1.2.4
	L-lactate dehydrogenase (cytochrome)	EC:1.1.2.3
	L-lactate dehydrogenase(cytochrome)	EC:1.1.1.27
	pyruvate dehydrogenase E1 component alpha subunit<br>pyruvate dehydrogenase E1 component beta subunit<br>pyruvate dehydrogenase E1 component	EC:1.2.4.1
Acetic acid biosynthesis	pyruvate dehydrogenase (quinone)	EC:1.2.5.1
	phosphate acetyltransferase	EC:2.3.1.8
	pyruvate oxidase	EC:1.2.3.3
	acetate kinase	EC:2.7.2.1
	acylphosphatase	EC:3.6.1.7
	acetyl-CoA hydrolase	EC:3.1.2.1
	propionate CoA-transferase	EC:2.8.3.1
	succinyl-CoA:acetate CoA-transferase	EC:2.8.3.18
	acetyl-CoA synthetase	EC:6.2.1.1
Phenylacetaldehyde biosynthesis	phenolic acid decarboxylase	EC:4.1.1.-
	aspartate aminotransferase, cytoplasmic<br>aspartate aminotransferase, mitochondrial	EC:2.6.1.1

	histidinol-phosphate aminotransferase	EC:2.6.1.9
	aromatic-amino-acid transaminase	EC:2.6.1.57
	cyclohexadienyl dehydratase	EC:4.2.1.51
PGI pathway	glycolate oxidase	EC:1.1.3.15
Malic acid and citric acid biosynthesis	pyruvate carboxylase	EC:6.4.1.1
	malate dehydrogenase	EC:1.1.1.37
	malate dehydrogenase (quinone)	EC:1.1.5.4
	citrate synthase	EC:2.3.3.1
	ATP citrate (pro-S)-lyase	EC:2.3.3.8
Sulfide biosynthesis	sulfite reductase (NADPH) flavoprotein alpha-component	EC:1.8.1.2
	aspartate aminotransferase	EC:2.6.1.1
	aspartate aminotransferase, cytoplasmic	
	aspartate aminotransferase, mitochondrial	
	methionine-gamma-lyase	EC:4.4.1.11

Reference

[1] N.R.A.D.T. Hofmann, Quantitative Studies, Taste Reconstitution, and Omission Experiments on the Key Taste Compounds in Morel Mushrooms (Morchella deliciosa Fr.), Journal of Agricultural & Food Chemistry, (2006). https://doi.org/10.1021/jf053131

[2] W. Ding, X. Ye, X. Zhao, et al., Fermentation characteristics of Pixian broad bean paste in closed system of gradient steady-state temperature field, Food Chemistry, 374 (2022) 131560. https://doi.org/10.1016/j.foodchem.2021.131560
